# Defining compartmentalized stem cell populations with distinct cell division dynamics in the ocular surface epithelium

**DOI:** 10.1242/dev.197590

**Published:** 2020-12-16

**Authors:** Ryutaro Ishii, Hiromi Yanagisawa, Aiko Sada

**Affiliations:** 1 Life Science Center for Survival Dynamics, Tsukuba Advanced Research Alliance (TARA), University of Tsukuba, Tsukuba 305-8577, Japan; 2 Graduate School of Comprehensive Human Sciences, University of Tsukuba, Tsukuba 305-8577, Japan; 3 Faculty of Medicine, University of Tsukuba, Tsukuba 305-8577, Japan; 4 International Research Center for Medical Sciences (IRCMS), Kumamoto University, Kumamoto 860-0811, Japan

**Keywords:** Ocular surface epithelium, Limbal stem cells, Label-retaining cell, Lineage tracing, Conjunctiva, Stem cell compartment, Mouse

## Abstract

Adult tissues contain label-retaining cells (LRCs), which are relatively slow-cycling and considered to represent a property of tissue stem cells (SCs). In the ocular surface epithelium, LRCs are present in the limbus and conjunctival fornix; however, the character of these LRCs remains unclear, owing to lack of appropriate molecular markers. Using three CreER transgenic mouse lines, we demonstrate that the ocular surface epithelium accommodates spatially distinct populations with different cell division dynamics. In the limbus, long-lived Slc1a3^CreER^-labeled SCs either migrate centripetally toward the central cornea or slowly expand their clones laterally within the limbal region. In the central cornea, non-LRCs labeled with Dlx1^CreER^ and K14^CreER^ behave as short-lived progenitor cells. The conjunctival epithelium in the bulbar, fornix and palpebral compartment is regenerated by regionally unique SC populations. Severe damage to the cornea leads to the cancellation of SC compartments and conjunctivalization, whereas milder limbal injury induces a rapid increase of laterally expanding clones in the limbus. Taken together, our work defines compartmentalized multiple SC/progenitor populations of the mouse eye in homeostasis and their behavioral changes in response to injury.

## Abstract (precis)

**Summary:** This study provides genetic tools to precisely mark and examine the dynamic behavior of heterogeneous stem cell populations in the ocular surface epithelium.

## INTRODUCTION

Tissue stem cells (SCs) play an important role in homeostasis and injury repair. Adult epithelial tissues – such as the skin, eye, oral mucosa and intestine – show proliferative heterogeneity. Infrequently dividing or ‘slow-cycling’ cells in the bulge region of skin hair follicles have been identified as label-retaining cells (LRCs) by DNA analog pulse-chase experiments (Bickenbach, 1981). The hierarchical stem/progenitor model – in which slow-cycling LRCs show unique, long-lived stem cell properties and give rise to short-lived, fast-dividing progenitors – has been applied to various epithelial and non-epithelial tissues (Bickenbach, 1981; Cotsarelis et al., 1990; Foudi et al., 2009; Fuchs, 2009; Mascr et al., 2012; Sánchez-Danés et al., 2016; Sangiorgi and Capecchi, 2008; Tumbar et al., 2004; Wilson et al., 2008). However, recent studies have challenged the universality of the hierarchical model, suggesting that the relationship between LRCs and their SC potential can be tissue- or context-dependent. In the interfollicular epidermis and oral epithelium, different epithelial compartments accommodate heterogeneous populations of SCs that show differences in cell division dynamics, location, molecular properties, biological functions and tumorigenic abilities (Byrd et al., 2019; Füllgrabe et al., 2015; Gomez et al., 2013; Kretzschmar et al., 2016; Page et al., 2013; Roy et al., 2016; Sada et al., 2016; Sánchez-Danés et al., 2016; Wang et al., 2020). In contrast, the single population model suggests that epithelial tissues are maintained not by functionally discrete SC populations, but by a homogeneous population of SCs that undergoes stochastic divisions and fate choices (Clayton et al., 2007; Doupé et al., 2012, 2010; Jones et al., 2019; Krieger and Simons, 2015; Piedrafita et al., 2020; Rompolas et al., 2012).

The ocular surface epithelium consists of the cornea and the conjunctiva and protects the eye from environmental damage. The cornea is covered by stratified, non-keratinizing squamous epithelium, which lies on the avascular corneal stroma. The conjunctival epithelium is comprised of three parts (bulbar, fornix and palpebral conjunctiva) and provides mucins required for the maintenance of the tear film (Hertsenberg and Funderburgh, 2015; Lavker et al., 2004). Severe corneal injury and loss of stem cells leads to an invasion of conjunctival cells to the cornea (conjunctivalization), resulting in the corneal opacity associated with neovascularization and eventually vision loss.

Pulse-chase experiments using histone H2B-GFP, BrdU or tritiated thymidine have suggested the existence of LRCs in the limbus and fornix regions of the conjunctiva (Cotsarelis et al., 1989; Parfitt et al., 2015; Wei et al., 1995). It has been proposed that the limbus contains a unique SC population known as limbal epithelial SCs, which give rise to progenitors that migrate toward the central cornea (Cotsarelis et al., 1989; Lavker and Sun, 2003). Limbal epithelial SCs have shown holoclone (i.e. the putative stem cell colonies)-forming ability *in vitro* (Ebato et al., 1988; Pellegrini et al., 1999) and are used for regenerative therapy of the corneal epithelium (Rama et al., 2010). The limbal SC model is supported by lineage tracing studies using inducible Cre-mediated labeling. Cells marked with K14^CreER^ or CAGG^CreER^ were observed migrating centripetally from the limbus to the central cornea (Amitai-Lange et al., 2015; Di Girolamo et al., 2015; Dorà et al., 2015; Richardson et al., 2017, 2016). Bmi1^CreER+^ can be used to label comparatively shorter-lived progenitor populations located in the central cornea (Kalha et al., 2018).

An alternative model, the corneal epithelial SC hypothesis, suggests the existence of SCs in the central cornea. In support of this model, a previous study demonstrated that corneal epithelial SCs that were transplanted to the limbus migrated to the central cornea when the entire cornea was removed (Majo et al., 2008). In addition, these corneal epithelial cells exhibited the ability to undergo serial transplantation, suggesting that self-renewing SCs exist in the entire corneal epithelium. K14^CreER^-based lineage tracing studies and transplantation and culture experiments also support the existence of an SC/progenitor population in the central or entire cornea (Amitai-Lange et al., 2015; Li et al., 2017). However, the lack of definitive regional markers to use as lineage tracing tools to specifically mark epithelial subpopulations has posed challenges for the determination of SC identity in the corneal epithelium.

Despite accumulating knowledge on corneal regeneration, the characteristics of conjunctival SCs has been insufficiently explored (Ramos et al., 2015). A theory of conjunctival transdifferentiation proposed that conjunctival epithelial cells may migrate to become the corneal epithelium (Shapiro et al., 1981). In contrast, more recent studies have shown that conjunctival and corneal epithelial cells exhibit distinct intrinsic properties and differentiation potential in the same environmental conditions, ruling out the possibility of conjunctival transdifferentiation (Cho et al., 1999; Wei et al., 1996, 1993). Based on the location of LRCs and their *in vitro* holoclone-forming ability, the fornix conjunctiva has been proposed to contain conjunctival epithelial SCs (Wei et al., 1995, 1993). Other studies have instead suggested the bulbar conjunctiva (Budak et al., 2005; Pellegrini et al., 1999) and palpebral conjunctiva (Chen et al., 2003) as epithelial SC locations. Given the lack of genetic mouse tools, there is little *in vivo* evidence addressing which cell populations of the conjunctival epithelium act as SCs and what lineage relationships exist among the three regions of the conjunctiva (bulbar, fornix and palpebra).

Studies in skin and other epithelial tissues have shown that epithelial SCs display plasticity in response to tissue damage and can change their lineages transiently or permanently (Belokhvostova et al., 2018; Dekoninck and Blanpain, 2019). In eyes, Nasser et al. combined K14^CreER^ with K15^GFP^ reporter and proposed that limbus epithelium deletion is repaired by dedifferentiation of corneal committed cells (Nasser et al., 2018). In contrast, the cornea is covered with conjunctiva-like epithelium after chemical burn or whole cornea epithelium deletion (Afsharkhamseh et al., 2016; Saika et al., 2005; Wei et al., 1995). How different subpopulations of SCs in the cornea (central versus peripheral) and conjunctiva (bulbar versus fornix versus palpebra) react to different levels of tissue damage remains unaddressed.

Previous studies have demonstrated that the limbus is molecularly defined by a high level of p63 (Trp63), K15 (Krt15) and Abcb5 (Pellegrini et al., 2001; Sartaj et al., 2017). Recent RNA sequencing studies have provided the whole transcriptome of H2B-GFP LRCs (Sartaj et al., 2017) and the entire ocular surface epithelium by single cell analysis (Altshuler et al., 2020; Kaplan et al., 2019). However, no definitive markers have been identified that faithfully label and distinguish the limbus from other populations of corneal epithelium for lineage tracing studies. We have previously identified two markers – Dlx1 and Slc1a3 – that preferentially label LRC SCs and non-LRC SCs, respectively, in the interfollicular epidermis of the skin (Sada et al., 2016). These two populations of SCs are largely independent of one another during homeostasis, but they also show the capacity to contribute to each other's lineage in response to injury. It remains unknown whether such SC compartments also exist in the cornea or conjunctiva. Here, we applied lineage tracing tools including Dlx1^CreER^, Slc1a3^CreER^ and K14^CreER^ to the ocular surface epithelium and characterized cellular dynamics under conditions of both homeostasis and injury. We showed that each distinct Cre-labeled population of stem and progenitor cells were highly compartmentalized and had different cell division dynamics. Under physical or chemical damage, these territorial segregations were lost, and SC lineages were altered. These findings provide new insight into the biological nature of ocular epithelial SCs.

## RESULTS

### LRCs and non-LRCs in the ocular surface epithelium can be identified by distinct CreER tools: K14^CreER^, Dlx1^CreER^ and Slc1a3^CreER^

**Fig. 1.**
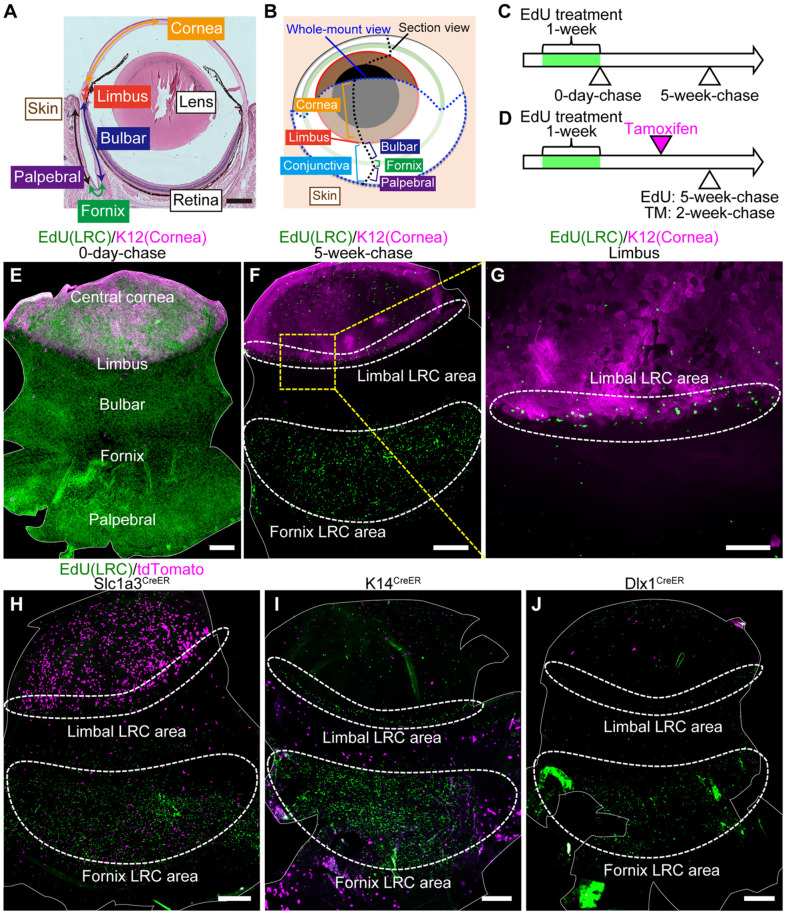
**Distinct CreER tools mark label-retaining cell (LRC) and non-LRC compartments.** (A) Hematoxylin-eosin stained mouse eye. (B) A schematic representation of the mouse eye. The eye was analyzed by sagittal sections (black dotted line) or whole-mount preparation of epithelial sheets (blue dotted line). (C) EdU pulse-chase scheme to detect LRCs in the ocular surface epithelium. (D) Scheme to examine the relationship of CreER^+^ cells with LRCs and non-LRCs. (E-G) Whole-mount staining of epithelial sheets after EdU pulse-chase experiments at 0-day-chase (E) and 5-week-chase (F,G). The solid white line outlines the whole-mount epithelial sheets (E,F). Limbal areas, surrounded by the yellow dashed square, are shown at a higher magnification in G. The white dashed line surrounds limbal and fornix LRC area. Green, EdU. Magenta, K12 (corneal marker). (H-J) Whole-mount staining of epithelial sheets after tamoxifen injection and EdU pulse-chase in Slc1a3^CreER^, K14^CreER^, and Dlx1^CreER^. The solid white line outlines the whole-mount epithelial sheets. The dashed white line surrounds the limbal and fornix LRC area. Green, EdU. Magenta, tdTomato. Scale bars: 500 μm (A,E,F,H-J); 200 μm (G).

LRCs have previously been shown to localize in the limbus and fornix conjunctiva (Parfitt et al., 2015). To evaluate the distribution of LRCs in the whole eye, we re-analyzed LRC locations by whole-mount staining of ocular epithelial sheets (Fig. 1A,B). A nucleotide analogue EdU was incorporated into all dividing cells, regardless of stem cell status, during week 1 of treatment (=pulse) (Fig. 1C). As the label is lost during divisions or differentiation, only cells that divide infrequently possess the marker after 5 weeks, and these are identified as LRCs. Mature adult mice were treated with EdU at 2- to 5-months of age, a period considered to be a steady-state homeostatic condition after intensive postnatal eye growth has ceased and before aging sets in (Kalha et al., 2018). The corneal and conjunctival epithelia were demarcated by K12 (Krt12) and K19 (Krt19), respectively (Fig. S1A-D) (Braun et al., 2003). At 0-day-chase, EdU+ cells were entirely distributed in the cornea and conjunctiva (Fig. 1E). At 5-week-chase, EdU+ cells were preferentially enriched in the limbus (which was at the boundary of K12-positive and -negative areas) and in the fornix area in the center of conjunctiva (Fig. 1F,G). This result confirmed the distribution pattern of LRCs in the ocular surface epithelium.

Next, we analyzed the relationship between LRC distribution and three CreER: K14^CreER^, Dlx1^CreER^ and Slc1a3^CreER^. According to previous reports using K14^CreER^-Confetti mice, K14^CreER^-labeled cells are uniformly distributed in the cornea and conjunctiva (Di Girolamo et al., 2015; Lobo et al., 2016). We used a different strain of K14^CreER^ with relatively weak K14 (Krt14) promoter activity and a low dose of tamoxifen to detect subpopulations of epithelial cells, as previously reported (Zhang et al., 2010). In addition to K14^CreER^, we used Dlx1^CreER^ and Slc1a3^CreER^, which we have previously established as SC markers in the skin interfollicular epidermis: Dlx1 marks LRCs and Slc1a3 marks non-LRCs (Sada et al., 2016). We used the same EdU pulse-chase condition to detect LRCs and injected tamoxifen at 2 weeks before analysis (Fig. 1D and Fig. S1E-G). No tdTomato reporter expression was observed without tamoxifen injection in any CreER used (Fig. S1H-J). We found Slc1a3^CreER^-labeled cells in the limbal LRC region as well as in the peripheral cornea, whereas K14^CreER^- and Dlx1^CreER^-labeled cells were preferentially located in the central cornea (Fig. 1H-J). In the conjunctiva, the LRC-dense fornix region was preferentially marked by Slc1a3^CreER^, and the bulbar and palpebral conjunctiva were marked by K14^CreER^. Although the labeling patterns of Slc1a3^CreER^ and Dlx1^CreER^ were opposite from what has been reported in the skin (Sada et al., 2016), these results suggest that Slc1a3^CreER^, Dlx1^CreER^ and K14^CreER^ can serve as useful genetic tools to distinguish cells within LRC and non-LRC regions in the cornea and conjunctiva.

To further address whether LRC and non-LRC compartments are molecularly defined by different markers, we stained epithelial sheets with antibodies that show preferential expression in different regions. Ifitm3, a marker of limbus (Altshuler et al., 2020), showed enriched expression in the LRC compartment, the limbus and fornix conjunctiva (Fig. S2A-E). In contrast, K14 and K13 (Krt13) were preferentially expressed in the non-LRC compartment in the cornea and conjunctiva (Fig. S2F-O). These results suggest that the ocular surface epithelium is heterogeneous with regards to cell division dynamics and molecular characters.

### Slc1a3^CreER^ marks limbal SC populations with two distinct dynamics

**Fig. 2.**
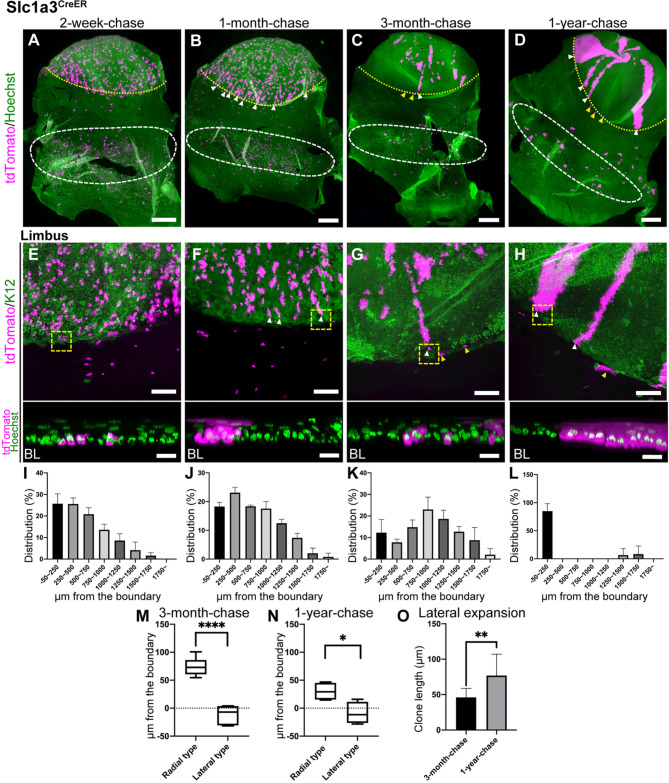
**Lineage tracing of Slc1a3^CreER^ in the limbus and cornea.** (A-H) Whole-mount immunostaining at 2-week-, 1-month-, 3-month- and 1-year-chases. The yellow dotted line represents the corneal/conjunctival boundary and the white dashed line represents tdTomato+ cell-enriched area in the fornix conjunctiva (A-D). Limbal areas are shown as a maximum-intensity projection (E-H, top). The representative limbal clones, surrounded by the yellow dashed square, are shown as a side view of *z*-stack confocal images (E-H, bottom; BL, basal layer). White arrowheads indicate tdTomato+ radial stripes extended from the limbus (B-D,F-H). Yellow arrowheads indicate tdTomato+ clones expanded laterally within the limbal region (C,D,G,H). Magenta, tdTomato. Green, Hoechst (A-D,E-H, bottom), K12 (E-H, top, corneal marker). (I-L) Distribution of the length of tdTomato+ clones from the corneal/conjunctival boundary at 2-week-, 1-month-, 3-month- and 1-year-chases expressed as the percentage of total clones. The boundary is defined by the K12 marker. *n*=3 mice*.* All tdTomato+ clones in whole-mount samples from a half eye were measured and used for quantification. Data are mean±s.d. (M,N) The positions of radial and lateral clones are measured from the boundary at 3-month-chase (M) and 1-year-chase (N). The boundary is defined by the K12 marker. *n*=3 mice*.* (O) Length of laterally expanding clones at 3-month-chase and 1-year-chase. *n*=3 mice*.* Data are mean±s.d. Box plots show median values (middle bars) and first to third interquartile ranges (boxes); whiskers indicate 1.5× the interquartile ranges. *P<0.05, **P<0.01, ****P<0.001 (Student's *t*-test). Scale bars: 500 μm (A-D); 200 μm (E-H, top); 20 μm (E-H, bottom).

To analyze the behavior of LRC population in the limbus, we used Slc1a3^CreER^ for lineage tracing. At 2-week-chase, tdTomato-labeled cells were predominantly observed in the limbus and peripheral cornea (Fig. 2A,E and Fig. S3A,E). To quantitatively analyze the distribution of labeled clones, we measured the length between the corneal/conjunctival boundary and the proximal edge of each clone. This was plotted in a histogram (Fig. S1K). The boundary was determined by K12 staining of whole-mount images. We found that the localization of Slc1a3^CreER+^ clones at 2-week-chase was highest within ∼500 µm from the corneal/conjunctival boundary, with a gradual decline toward the central cornea (Fig. 2I). By 1-month-chase, the labeled cells started to show radial stripes, indicating the continuous migration and expansion of cells from the limbus toward the central cornea, as previously reported (Fig. 2B,F,J and Fig. S3B,F) (Amitai-Lange et al., 2015; Di Girolamo et al., 2015; Dorà et al., 2015; Richardson et al., 2017, 2016). The limbal clones were found in the basal layer at the beginning of chase (Fig. 2E, bottom). These clones reached the upper-most layers of the epithelium after 1-month-chase, an indication of their differentiation ability (Fig. 2F, bottom). At 3-month-chase, the distribution of clones shifted toward the central cornea and peaked ∼750-1000 µm from the corneal/conjunctival boundary (Fig. 2C,G,K and Fig. S3C,G). This indicates that some clones were short-lived and lost within a few months.

Notably, the labeled cells in the limbal region showed two distinct behaviors: (1) the radial stripes extended from the limbus (Fig. 2C,G, white arrowheads, radial stripe type) and (2) the clones expanded within the limbal region (Fig. 2C,G, yellow arrowheads, lateral-expansion type). Quantification of the position of the radial and lateral clones showed that the former were located at the K12-positive corneal area, whereas the latter were located at the boundary or K12-negative area (Fig. 2M). At 1-year-chase, both radial and lateral clones were maintained and located in spatially separated compartments (Fig. 2D,H,L,N and Fig. S3D,H-N). The lateral clones became apparent after long-term chase and showed modest expansion over 1 year of chase, indicating their slow-cycling, infrequently dividing nature (Fig. 2O). Whole-mount staining with the vessel marker CD31 (Pecam1) showed that the K12-negative limbal region was enriched with smaller capillary vessels, whereas the K12-positive corneal region was generally avascular (Fig. S3O-Q). This indicates a possible role for distinct vascular environments in the regulation of radial and lateral clones. Taken together, Slc1a3^CreER^ lineage tracing studies suggest that the limbal LRC region contains long-lived SCs that undergo either centripetal migration to replenish the corneal epithelium or lateral expansion to maintain the limbal compartment with relatively slow turnover.

### K14^CreER+^ and Dlx1^CreER+^ short-lived progenitor populations in the central cornea

**Fig. 3.**
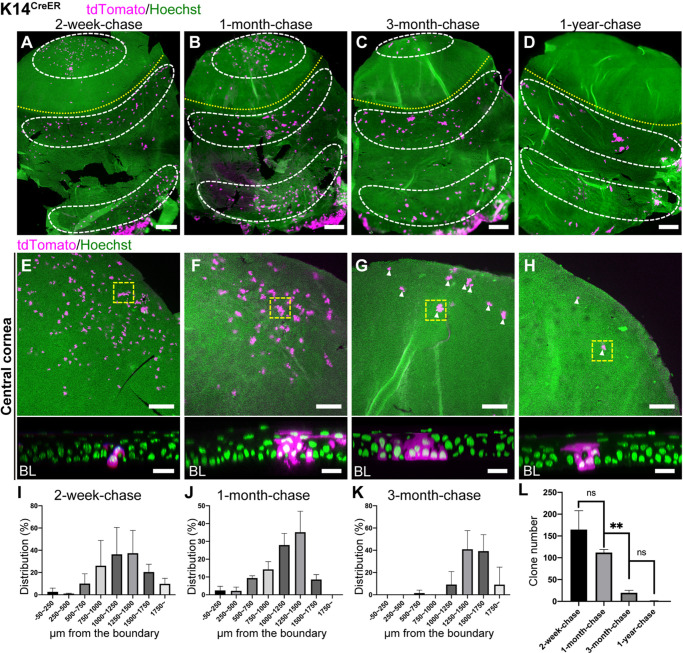
**Lineage tracing of K14^CreER^ in the cornea.** (A-H) Whole-mount immunostaining at 2-week-, 1-month-, 3-month- and 1-year-chases. The yellow dotted line represents the corneal/conjunctival boundary and the white dashed line indicates the tdTomato+ cell-enriched area (A-D). Central corneal areas are shown as a maximum-intensity projection (E-H, top). The representative central corneal clones, surrounded by the yellow dashed square, are shown as a side view of *z*-stack confocal images (E-H, bottom; BL, basal layer). Arrowheads indicate tdTomato+ cells (G,H). Magenta, tdTomato. Green, Hoechst. (I-K) Distribution of the length of tdTomato+ clones from the corneal/conjunctival boundary at 2-week-, 1-month- and 3-month-chases expressed as the percentage of total clones. The boundary is defined by the K12 marker. (L) The number of tdTomato+ clones per half whole-mount sample was quantified at the indicated time points. *n*=3 mice (I-L). All tdTomato+ clones in whole-mount samples from a half eye are measured and used for quantification. Data are mean±s.d. **P< 0.01 (one-way ANOVA followed by the Bonferroni test). ns, not significant. Scale bars: 500 μm (A-D); 200 μm (E-H, top); 20 μm (E-H, bottom).

To address whether cells in the central cornea retain SC properties *in vivo*, we traced the fate of K14^CreER^- and Dlx1^CreER^-marked cells, which were preferentially observed in non-LRCs in the central corneal region (Fig. 1I,J). With this tool, it is possible to determine whether labeled cells are able to self-maintain themselves for long periods of time (in accordance with the corneal epithelial SC hypothesis) or are instead supplied from limbal epithelial SCs. At 2-week-chase, K14^CreER+^ clones preferentially located in the basal layer of the central cornea (Fig. 3A,E and Fig. S4A,E). A quantitative analysis showed that these cells were primarily located 1000-1500 µm away from the corneal/conjunctival boundary (Fig. 3I), which is distinct from the distribution of Slc1a3^CreER+^ clones (Fig. 2I). During 1 to 3 months of chase, the clone distribution was slightly shifted toward the central cornea and peaked at ∼1500 µm away from the boundary (Fig. 3B,C,F,G,J,K and Fig. S4B,C,F,G). These observed clones in the central cornea remained until 3 months of chase, and the number of clones was markedly reduced after 1 year of chase (Fig. 3D,H,L and Fig. S4D,H). At 1-year-chase, remaining clones in the central cornea consisted of basal cells and a few suprabasal cells, which may reflect their limited ability to differentiate (Fig. 3H, bottom). Although the labeling efficiency of Dlx1^CreER^ is much lower than K14^CreER^, lineage tracing by Dlx1^CreER^ showed similar labeling patterns and cellular dynamics (Fig. S5). These results suggested that the central cornea (i.e. the non-LRC territory of the cornea) contained shorter-lived progenitor populations which were marked by K14^CreER^ and Dlx1^CreER^.

### Three distinct SC populations in the conjunctiva maintain their own compartments

**Fig. 4.**
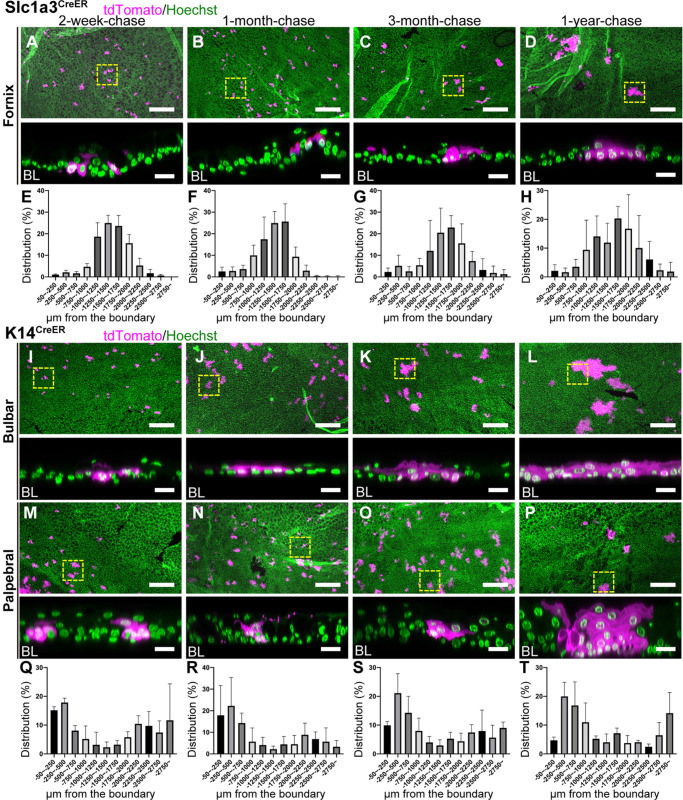
**Lineage tracing of Slc1a3^CreER^ and K14^CreER^ in the conjunctiva.** (A-D) Lineage tracing of Slc1a3^CreER^ in the conjunctiva at 2-week-, 1-month-, 3-month- and 1-year-chases. Fornix conjunctival areas are shown as a maximum-intensity projection (A-D, top). The representative fornix conjunctival clones, surrounded by the yellow dashed square, are shown as a side view of *z*-stack confocal images (A-D, bottom; BL, basal layer). (E-H) Distribution pattern of tdTomato+ clones. Length from the corneal/conjunctival boundary to the center of each clone was measured. The boundary is defined by the K12 marker. *n*=3 mice. All tdTomato+ clones in whole-mount samples from a half eye were measured and used for quantification. Data are mean±s.d. (I-P) Lineage tracing of K14^CreER^ in the conjunctiva at 2-week-, 1-month-, 3-month- and 1-year-chases. Bulbar (I-L) and palpebral (M-P) conjunctival areas are shown as a maximum-intensity projection (I-P, top). The representative bulbar and palpebral conjunctival clones, surrounded by the yellow dashed square, are shown as a side view of *z*-stack confocal images (I-P, bottom; BL, basal layer). Magenta, tdTomato. Green, Hoechst. (Q-T) Distribution pattern of tdTomato+ clones. Length from the corneal/conjunctival boundary to the center of each clone was measured. The boundary is defined by the K12 marker. *n*=3 mice. All tdTomato+ clones in whole-mount samples from a half eye were measured and used for quantification. Data are mean±s.d. Scale bars: 200 μm (A-D,I-P, top); 20 μm (A-D,I-P, bottom).

SC identity in the conjunctiva remains elusive. To define the locations of SCs in the conjunctiva and their cellular lineages, we used Slc1a3^CreER^ as a marker of the LRC region in the fornix and K14^CreER^ as a marker of the non-LRC region in the bulbar and palpebral conjunctiva (Fig. 1H,I). We first tested whether the Slc1a3^CreER+^ LRC population in the fornix act as SCs and which epithelial compartments are maintained by this population. At 2-week-chase, we found that the clones marked by Slc1a3^CreER^ in the fornix conjunctiva were located ∼1500 µm away from the corneal/conjunctival boundary toward the eyelid and consisted of a small cluster of basal cells (Figs 2A, 4A,E and Fig. S6A,E). These clones expanded in size and remained in the same region after 1 year of chase (Figs 2B-D, 4B-D,F-H and Fig. S6B-D,F-H). No apparent migration of cells was detected from the fornix conjunctiva to other regions, including the bulbar, palpebral conjunctiva and cornea, indicating that fornix LRCs are long-lived SCs that regenerate their own compartment. To further address which cells contribute to the regeneration of the bulbar and palpebral conjunctival epithelium, we performed lineage tracing using K14^CreER^, which preferentially labels non-LRCs in the bulbar and palpebral conjunctiva (Figs 3A, 4I,M,Q and Fig. S6I,M,Q,U). K14^CreER+^ showed double peaks of clone distribution ∼500 µm (bulbar conjunctiva) and ∼2500 µm (palpebral conjunctiva) away from the corneal/conjunctival boundary (Fig. 4Q). This is distinct from the localization observed with Slc1a3^CreER^ (Fig. 4E). After chase, K14^CreER+^ clones in the bulbar and palpebral conjunctiva showed no directed movement and expanded within their own territories (Fig. 4J-L,N-P,R-T and Fig. S6J-L,N-P,R-T,V-X). Overall, these results suggest that three distinct SC populations are located in the conjunctiva and maintained in their own compartments. Therefore, it is likely not the case that fornix LRCs are the sole source of SCs to reconstitute the entire conjunctiva or transdifferentiate into the corneal epithelium.

### Injury triggers remodeling of SC compartments in the ocular surface epithelium

**Fig. 5.**
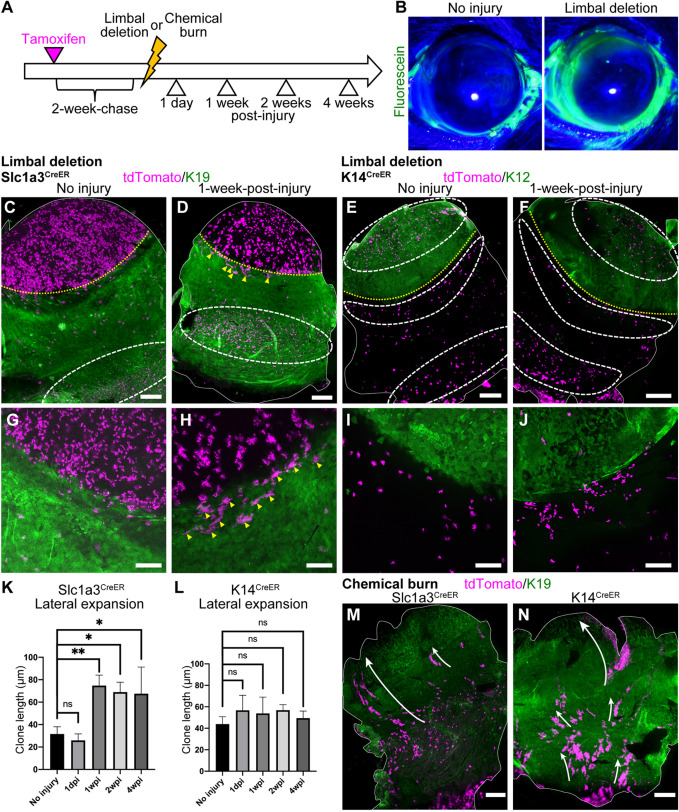
**Dynamics of Slc1a3^CreER^ and K14^CreER^ population after injury.** (A) Experimental scheme of injury experiments. (B) Fluorescein staining of eye before and after limbal deletion. (C-J) Whole-mount immunostaining after limbal epithelial deletion. Control (C,E,G,I) and 1-week-post-injury (D,F,H,J) are shown. The solid white line indicates the whole-mount epithelial sheets (C-F). The yellow dotted line indicates corneal/conjunctival boundary and the white dashed line indicates the tdTomato+ cell-enriched area (C-F). Yellow arrowheads indicate tdTomato+ clones expanding laterally within the K19-positive region (D,H). Magenta, tdTomato. Green, K19 (C,D,G,H, conjunctival marker), K12 (E,F,I,J, corneal marker). (K,L) Length of laterally expanding clones after limbal deletion in Slc1a3^CreER^ and K14^CreER^. *n*=3 mice. All tdTomato+ clones in whole-mount samples from a half eye were measured and used for quantification. (M,N) Whole-mount immunostaining 1 week after chemical burn. Slc1a3^CreER+^ (M) or K14^CreER+^ (N) cells are shown. The solid white line outlines the whole-mount epithelial sheets. The white arrow represents the movement of conjunctival tdTomato+ clones. Magenta, tdTomato. Green, K19 (conjunctival marker). Data are mean±s.d. *P<0.05, **P<0.01 (one-way ANOVA followed by Bonferroni test). ns, not significant. Scale bars: 500 μm (C-F,M,N); 200 μm (G-J).

It has been shown that epithelial SCs in the skin and eye have the potential to alter their behavior in a context-dependent manner. To test how different SC/progenitor populations in the cornea and conjunctiva respond to the injury, we applied two types of injury – limbal epithelial deletion and chemical burn – and analyzed the behavior of cells residing in the distinct epithelial compartments. Previous reports suggest that limbal SC deletion is recovered by de-differentiation and migration of progenitor cells located in the cornea (Nasser et al., 2018). We took advantage of Slc1a3^CreER^ and K14^CreER^ to distinguish cells in the limbus, peripheral, central cornea and different compartments of conjunctiva to determine which populations are the source of limbal regeneration. Two weeks after tamoxifen injection, we induced limbal epithelial injury by removing the epithelium located above the vessels using an ophthalmic rotating burr (Fig. 5A). The limbal epithelial deletion was confirmed by fluorescein staining (Fig. 5B). Before limbal deletion, Slc1a3^CreER+^ cells were located in the limbus and peripheral cornea (Fig. 5C,G). In contrast, K14^CreER+^ cells were located in the central cornea and the bulbar conjunctiva (Fig. 5E,I). Upon injury, Slc1a3^CreER+^ clones started to expand laterally in the K19-positive region (Fig. 5D,H,K and Fig. S7A-F, yellow arrowheads). These clones appeared quickly after the injury, and it is possible that this population corresponds to the laterally-expanding clones that we observed at 3 months and 1 year of chase during normal homeostasis (Fig. 2C,D,G,H, yellow arrowheads). At 4-weeks post-injury, the radial stripes reappeared at the K19-negative corneal region (Fig. S7C,F, white arrowheads). In contrast, K14^CreER+^ cells, both in the central cornea and the bulbar conjunctiva, showed no such behavior change upon injury (Fig. 5E,F,I,J,L and Fig. S7G-L). Thus, it appears that slow-cycling Slc1a3^CreER+^ cells in the K19-positive limbal compartment in the vicinity of injury were rapidly activated, contributing to the recovery of the limbal epithelium. In contrast, K14^CreER+^ cells in the central cornea and bulbar conjunctiva did not participate in the repair process. Our results suggest differences in behavior between Slc1a3^CreER^-marked (limbus/peripheral cornea) and K14^CreER^-marked (central cornea/bulbar conjunctiva) cell populations in response to limbal epithelial injury.

Finally, we induced chemical injury by applying sodium hydroxide solution to the cornea (Saika et al., 2005). Stromal injury by alkali burn leads to limbal SC deficiency, inducing conjunctivalization of the corneal surface and neovascularization (Joussen et al., 2003). However, it is unclear which conjunctival population responds to the corneal damage. In our study, after alkaline burn, the cornea was largely covered by the conjunctival cells, which expressed the conjunctival marker K19 in the entire epithelium and lost K12 expression (Fig. 5M,N and Fig. S7M-P). By using Slc1a3^CreER^, we found that epithelial SCs in the fornix conjunctiva migrated to the corneal area and started to express K19 (Fig. 5M). This lineage disruption persisted for 2 weeks (Fig. S7M,N). Similarly, K14^CreER+^ clones, both in the bulbar and palpebral conjunctiva, migrated toward the cornea (Fig. 5N and Fig. S7O,P). These observations indicate that conjunctival SCs change their propensity for differentiation and tissue coverage after the extensive damage in order to compensate for the loss of limbal SCs.

## DISCUSSION

**Fig. 6.**
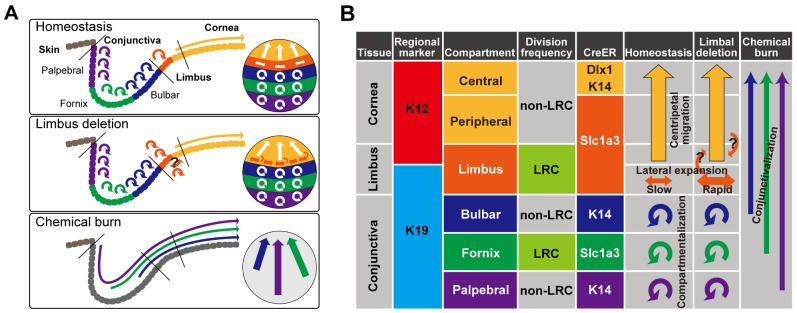
**Proposed model of compartmentalized stem and progenitor populations with distinct cell division dynamics in the ocular surface epithelium.** (A) Diagram representing the compartmentalization of the ocular surface epithelium and SC dynamics in homeostatic and post-injury conditions. (B) Summary table of genetic markers to define distinct SC compartments and lineage relationships. The long arrows represent the migration of cells from one compartment to another. The round arrows represent self-maintenance of each compartment by local SCs.

Our work provides genetic tools to precisely mark and examine the dynamic behavior of multiple SC/progenitor populations in the ocular surface epithelium during homeostasis and injury repair, and to molecularly characterize each population. The characteristics of slow-cycling cells in the ocular surface epithelium have been difficult to ascertain given a lack of definitive molecular markers and lineage tracing mouse models. In the present study, we took advantage of three CreER tools and analyzed the cellular dynamics of the corneal and conjunctival epithelium during homeostasis and injury repair (Fig. 6). We identified distinct compartments in the ocular surface epithelium, characterized by anatomical location, marker expression and cell division dynamics. The Slc1a3^CreER^ marker preferentially labels LRC regions in the limbus and fornix conjunctiva, and has a distinct labeling pattern compared with that of the K14^CreER^ or Dlx1^CreER^ lines. Notably, three compartments in the conjunctiva – the bulbar, fornix and palpebral conjunctiva – are governed by local SC populations marked by distinct CreER tools, indicating differences in molecular properties. Chemical burn triggers disruption of these SC compartments and invasion of all three conjunctival SC populations into the corneal region. The mechanism underlying the territorial segregation of epithelial SCs is unknown, but could potentially involve stromal architecture, vascular patterns, extracellular matrix or secreted factors.

Further, we found that Slc1a3^CreER^ activity is enriched in LRC regions of the ocular surface epithelium, but the functional importance of Slc1a3 remains to be addressed. The *Slc1a3* gene encodes a glutamate/aspartate transporter that is involved in glutamatergic neurotransmission in the brain (Kanai and Hediger, 2004). In peripheral tissues, Slc1a3 is widely expressed in epithelial cells (Berger and Hediger, 2006). Slc1a3 is upregulated in actively cycling SCs of the skin interfollicular epidermis, hair follicles and sebaceous glands and plays a role in SC/progenitor cell activation (Reichenbach et al., 2018; Sada et al., 2016) and promotes cell proliferation and survival of cancer cells under conditions of nutrient starvation or hypoxia (Garcia-Bermudez et al., 2018; Tajan et al., 2018). It also mediates tumor growth by exchanging glutamate and aspartate between squamous cell carcinoma and carcinoma-associated fibroblasts in a stiff environment (Bertero et al., 2019). Given that the proliferative heterogeneity of SCs is highly correlated with metabolic regulation (Coller, 2019; Coloff et al., 2016), the roles of Slc1a3 and amino acid metabolism in different SC niches of the ocular surface epithelium are important topics to address.

The Slc1a3^CreER+^ SC population in the limbus showed two distinct behavioral patterns: migration centripetally toward the central cornea and expansion laterally within the limbal compartment. Our analysis cannot fully address whether these two types of Slc1a3^CreER+^ clones represent two discrete SC populations or rather differences in cell division pattern (asymmetric versus symmetric) of the same SC population. During steady-state tissue turnover, the dynamics of the limbal SCs is biased toward the production of corneal epithelial progenitors in the central cornea. Conditions of limbal injury, however, shift the dynamics toward the limbal-expansion mode and induce rapid expansion of the Slc1a3^CreER+^ population within the limbus. Therefore, the Slc1a3^CreER+^ population in the limbus, despite being relatively slow-cycling in nature, appears to equip itself with a back-up system to quickly respond to tissue damage. Our findings do not rule out the possibility that de-differentiation of peripheral corneal progenitor cells may contribute to limbal SC regeneration. To address this issue, further studies are needed that track the Slc1a3^CreER+^ population using different methods, for example live imaging during the process of injury repair.

The K14^CreER^ line used in our study marked subpopulations of ocular surface epithelium, which differed from the previously reported K14^CreER^ pattern with more uniform labeling (Amitai-Lange et al., 2015; Di Girolamo et al., 2015; Richardson et al., 2017). In our previous work, use of a less efficient K14^CreER^ line allowed us to achieve enriched labeling of the basal layer of the interfollicular epidermis to identify a subpopulation of epithelial cells with relatively higher K14 promoter activity (Sada et al., 2016; Zhang et al., 2010). The differences in the K14^CreER^ labeling pattern may also be attributed to the Cre reporter line used in our studies: Rosa-tdTomato shows higher activity than other reporters, including Confetti. Our results suggest that the combination of K14^CreER^ with a Cre reporter line may be an effective tool to distinguish different compartments of cornea and conjunctiva.

The SC potential of the central cornea is a subject of debate. Our lineage tracing study showed that K14^CreER+^ and Dlx1^CreER+^ cells in the central corneal compartment are mostly shorter-lived, which might fit the definition of progenitor cells. However, the rare existence of long-lived populations in the central cornea may reflect the SC leakage phenomenon (Lobo et al., 2016).

After the damage of SCs and their niches, the SC compartment is remodeled according to the severity of the injury (i.e. localized versus diffuse, superficial versus deep), and different SC populations react differently to repair damaged epithelium. We demonstrated that Slc1a3^CreER+^ limbal/peripheral corneal populations and K14^CreER+^ central corneal populations responded differently to the limbal deletion, possibly owing to the differences in their intrinsic properties or external conditions along the spherical axis of the ocular epithelium. In particular, we identified a previously uncharacterized Slc1a3^CreER+^ slow-cycling population of cells in the limbus. This population was inactive during normal homeostasis but rapidly expanded in response to injury, a useful property for limbal regeneration. Our K14^CreER^ lineage tracing study also demonstrated that conjunctival SCs in the bulbar region, even though they are located closely to the limbus, do not respond to the limbal epithelial damage. This supports the idea of distinct patterns of response to limbal deletion between corneal and conjunctival SCs. In contrast, chemical burn, an experimental model of limbal SC deficiency, triggers invasion of conjunctival SC populations to the cornea without adaptation of their fates to corneal lineages. It is plausible that the limbal SCs may play an inhibitory role for conjunctival SCs to enter the corneal compartment during homeostasis, whereas elimination of limbal SCs by chemical injury might lead to conjunctivalization of the eye. Thus, our data reveal functional cross-interaction between SC types in the ocular epithelium and demonstrate their plasticity in response to tissue damage.

Studies to define and control corneal and conjunctival SCs would be of great clinical value for the treatment of extensive ocular injuries such as severe chemical burns or inflammatory diseases, e.g., Stevens-Johnson syndrome (Barut Selver et al., 2017). Our genetic tools can be used to further investigate the cellular and molecular mechanisms of SC plasticity in different disease or injury models *in vivo* and to identify potential therapeutic strategy for limbal SC deficiency. Given that SC heterogeneity is associated with differential tumorigenic ability, regenerative capacity and interaction with non-epithelial cell types in skin (Rognoni and Watt, 2018), future studies should further unravel the biological significance of multiple SC/progenitor populations in the ocular surface epithelium and their specific roles in different physiological conditions.

## MATERIALS AND METHODS

### Mice

All mouse experiments were performed according to the protocols approved by the Animal Care and Use Committee of the University of Tsukuba and Kumamoto University. Mice were housed in the Laboratory Animal Resource Center at the University of Tsukuba and the Center for Animal Resources and Development at Kumamoto University. For lineage tracing, Slc1a3^CreER^ (C57BL/6J) (Nathans, 2010) (The Jackson Laboratory, 012586), Dlx1^CreER^ (C57BL/6J) (Taniguchi et al., 2011) (The Jackson Laboratory, 014551) and K14^CreER^ (mixed background of CD1 and C57BL/6J) (Vasioukhin et al., 1999) (a gift from E. Fuchs, Rockefeller University, New York, NY, USA) were crossed with Rosa-tdTomato reporter mice (C57BL6/J) (Madisen et al., 2010) (The Jackson Laboratory, 007905). C57BL/6J wild-type mice were purchased from Charles River Laboratories or Japan SLC. All experiments involved mature adult male and female mice aged 2 to 5 months and in steady-state homeostasis.

### EdU and tamoxifen treatment

To label LRCs, mice were injected intraperitoneally with 50 μg/g body weight EdU (Invitrogen) twice per day for 1 week, followed by 5 weeks of chase without EdU before the animals were sacrificed. For lineage tracing using K14^CreER^, mice were injected intraperitoneally with a single dose of tamoxifen (50 μg/g body weight; Sigma-Aldrich) at 2-3 months of age. For Slc1a3^CreER^ and Dlx1^CreER^ lines, mice were injected with tamoxifen (100 μg/g body weight) for 5 consecutive days. Mice were sacrificed at 2-week-, 1-month-, 3-month- and 1-year-chases after the last injection. CreER/Rosa-tdTomato mice without tamoxifen injections were used to examine the leakiness of Cre. All treatment was started in mature adult mice aged between 2 and 5 months.

### Staining of eye sections

Enucleated eyes were fixed in 4% paraformaldehyde (PFA) overnight and snap-frozen in OCT compound (Tissue-Tek). For histological analysis, 10-µm sections were air dried and washed in PBS, followed by staining with Hematoxylin (Wako, 131-09665) for 20 min and Eosin Y (Wako, 058-00062) for 15 s. Sections were dehydrated and mounted in Entellan new mounting solution (Merck Millipore, HX73846161). For immunostaining, frozen sections were incubated with blocking solution (2.5% donkey serum and 2.5% goat serum in PBS) for 1 h at room temperature. Primary antibodies were used at the following dilutions: rabbit anti-K12 (1:100, Abcam, ab185627) and rabbit anti-K19 (1:100, Abcam, ab52625). Secondary antibodies (Alexa 488 or 546, Invitrogen) were used at 1:200 dilution. All samples were counterstained with Hoechst (Sigma-Aldrich, B2261) for 10 min and mounted. Preparations were analyzed and imaged using a Zeiss Axio Imager.Z2. The brightness and contrast of images were adjusted with equal intensity among different experimental groups of mice using Adobe Photoshop.

### Whole-mount immunostaining

Eyes were cut in half and incubated in EDTA (20 mM)/PBS in an orbital shaker at 37°C for 2 h to separate the epithelium from the mesenchyme as an intact sheet. Epithelial sheets were fixed in 4% PFA overnight at 4°C with gentle shaking. The epithelial sheets were washed, incubated in blocking buffer (1% bovine serum albumin, 2.5% donkey serum, 2.5% goat serum, 0.8% Triton in PBS) for 3 h at room temperature, and incubated with primary antibodies and blocking buffer overnight at room temperature. Samples were then washed four times in 0.2% Tween/PBS for 1 h at room temperature and incubated overnight with secondary antibodies at 4°C. After washing, samples were counterstained with Hoechst (Sigma-Aldrich, B2261) for 1 h and mounted. Primary antibodies were used at the following dilutions: rabbit anti-K12 (1:300, Abcam, ab185627), rabbit anti-K19 (1:300, Abcam, ab52625), rabbit anti-K14 (1:100, BioLegend, 905304), anti-Fragilis (Ifitm3; 1:100, Abcam, ab15592) and rabbit anti-K13 (1:100, Abcam, ab92551). Secondary antibodies (Alexa 488, 546 or 647, Invitrogen) were used at 1:200 dilution.

For EdU staining, the epithelial sheets were blocked in blocking buffer for 3 h at room temperature and incubated with the Alexa Fluor 488 Click-iT EdU Imaging Kit (Invitrogen) for 1 h at room temperature. Samples were further washed three times for 15 min in 0.2% Tween/PBS at room temperature. Samples were co-stained with primary and secondary antibodies as described above. To recover tdTomato fluorescence, samples were incubated three times in 0.1 M EDTA for 20 min, followed by a 5-min wash in PBS before mounting.

For whole-mount staining including stroma, the eyeball was enucleated and the anterior part of the eye (from the cornea to the bulbar conjunctiva, including the sclera) was separated from the posterior part. The iris and lens were removed carefully. Subsequently, the collected samples were fixed in 4% PFA overnight at 4°C. After blocking, samples were stained with the following primary antibodies: rabbit anti-K12 (1:300, Abcam, ab185627) and anti-CD31 (1:100, BD Biosciences, 550274). Secondary antibodies (Alexa 488, 546 or 647, Invitrogen) were used at 1:200 dilution. Finally, samples were slit in a radial fashion so they could be mounted flat in antifade mounting medium. Whole-mount preparations were analyzed and imaged using a Zeiss Axio Imager.Z2 or confocal microscope (LSM710). All confocal data are shown as projected *z*-stack images.

### Injury

Limbal deletion and chemical injury were performed as previously described (Nasser et al., 2018). For all injury experiments, mice were anesthetized with tribromoethanol. Mice were intraperitoneally injected with carprofen (5 mg/kg) and monitored for pain and eye infections during and after injury procedures. The limbus epithelium located above the vessels of the stroma was removed using an ophthalmic rotating burr (Alger, AlgerBrush II BR2-5) under a stereo microscope (Zeiss). To verify the complete removal of the epithelium, a drop of 1 mg/ml fluorescein sodium (Sigma Aldrich, F6377) was applied to the cornea. For chemical injury, 3 µl of 1 N sodium hydroxide solution was applied to the cornea. Subsequently, the eye was washed with PBS.

### Quantification of microscope images

The length between the corneal/conjunctival boundary and the proximal edge of each clone was measured in the whole-mount samples using ImageJ software. Distribution percentages were calculated using GraphPad Prism 8 (GraphPad Software). All quantifications for Slc1a3^CreER^ and K14^CreER^ lineage tracing were independently performed on three mice. Owing to low label efficiency of Dlx1^CreER^, the following numbers of mice were used for quantification at each chase period: three mice at 2-week-chase, three mice at 4-week-chase, seven mice at 3-month-chase and six mice at 1-year-chase. All tdTomato+ clones in whole-mount samples from a half eye of each mouse were measured. Data are mean±s.d.

### Reproducibility

All experiments were independently performed at least three times with different mice, and the representative images or an average data are shown.

## Supplementary Material

Supplementary information

Reviewer comments
