# Supplementary material for: Defining compartmentalized stem cell populations with distinct cell division dynamics in the ocular surface epithelium
Source: Development. 2020 Dec 16;147(24):dev197590. doi: 10.1242/dev.197590 (PMC7758628; doi:10.1242/dev.197590)
Supplement: Supplementary information [file develop-147-197590-s1.pdf]

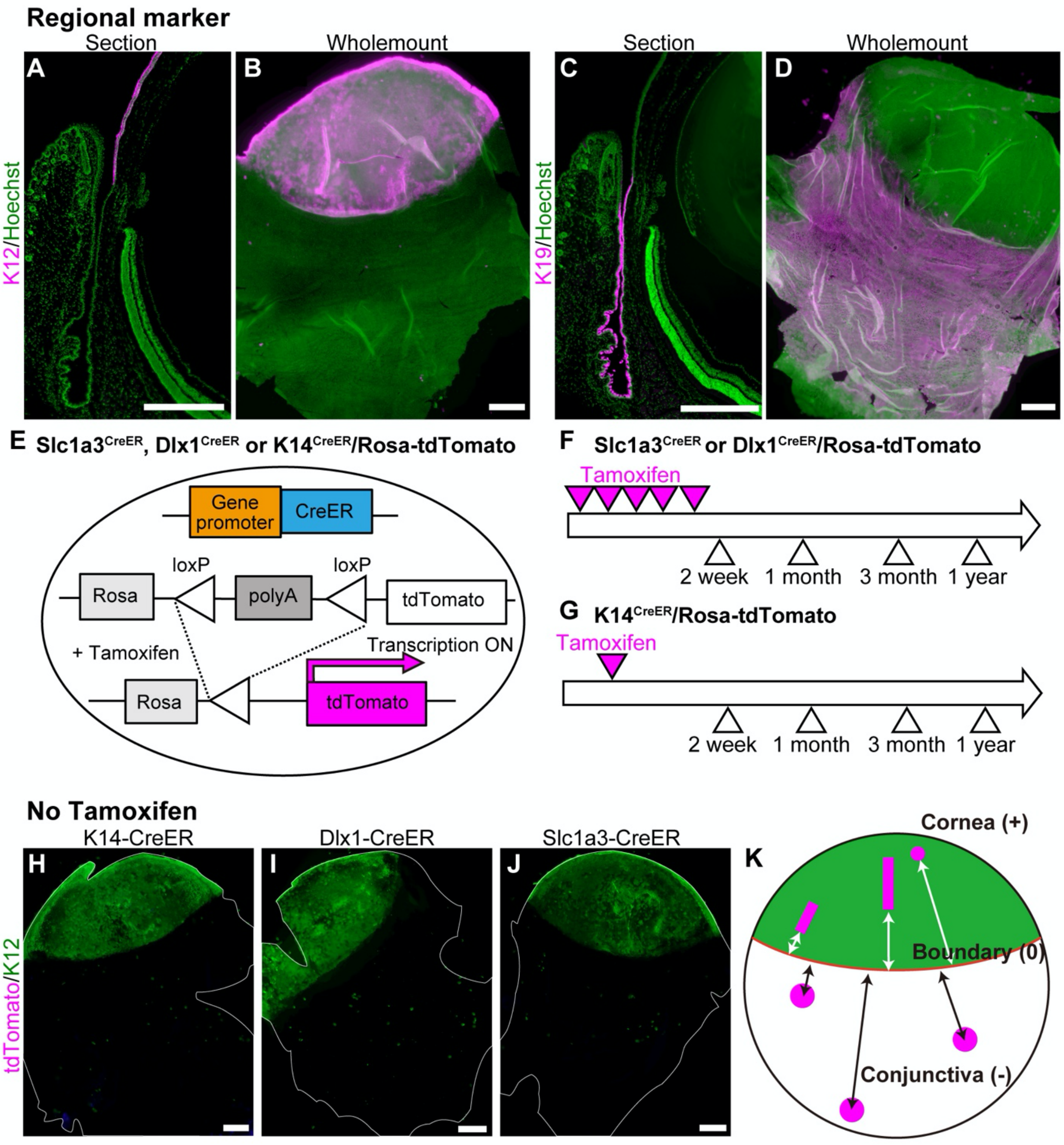

Figure S1. Tamoxifen-inducible Cre for cell fate tracking. (**A-D**) Immunostaining of wild-type mouse eye on section (A, C) or whole-mounts (B, D). K12 and K19 (magenta) were used as markers of the corneal and conjunctival epithelium, respectively. Hoechst nuclear staining in green. Scale bars: 500 µm. (**E**) Schematic representation of the tamoxifen-inducible CreER system. (**F, G**) Scheme for long-term lineage tracing. Schedule of tamoxifen injection (magenta arrowheads) and tissue collection (white arrowheads) is shown. (**H-J**) K14^{CreER}, Dlx1^{CreER} and Slc1a3^{CreER} without tamoxifen injection. The white line outlines the whole-mount epithelial sheets. Magenta, tdTomato. Green, K12 (corneal marker). Scale bars: 500 µm. (**K**) Diagram shows the measurement of the length between the peripheral edge of the tdTomato+ clone and the corneal/conjunctival boundary.

Development • Supplementary information

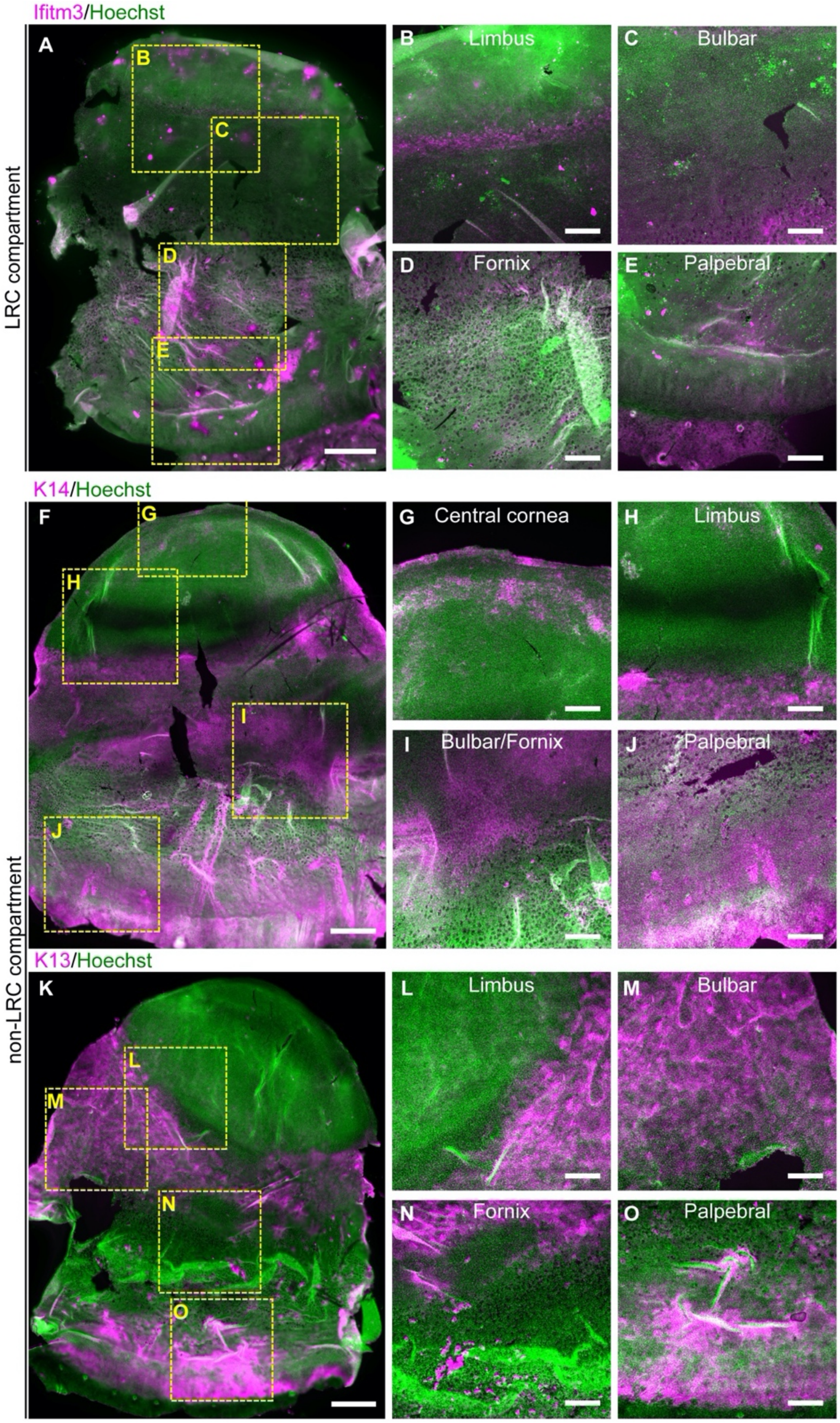

Figure S2. Regional markers to define distinct compartments in the ocular surface epithelium. (**A-O**) A whole-mount staining of epithelial sheets. Areas surrounded by the yellow dashed square are shown with higher magnification (B-E, G-J, L-O). Magenta, Ifitm3 (A-E), K14 (F-J) and K13 (K-O). Green, Hoechst. Scale bars: 500 µm (A, F, K), 200 µm (B-E, G-J, L-O).

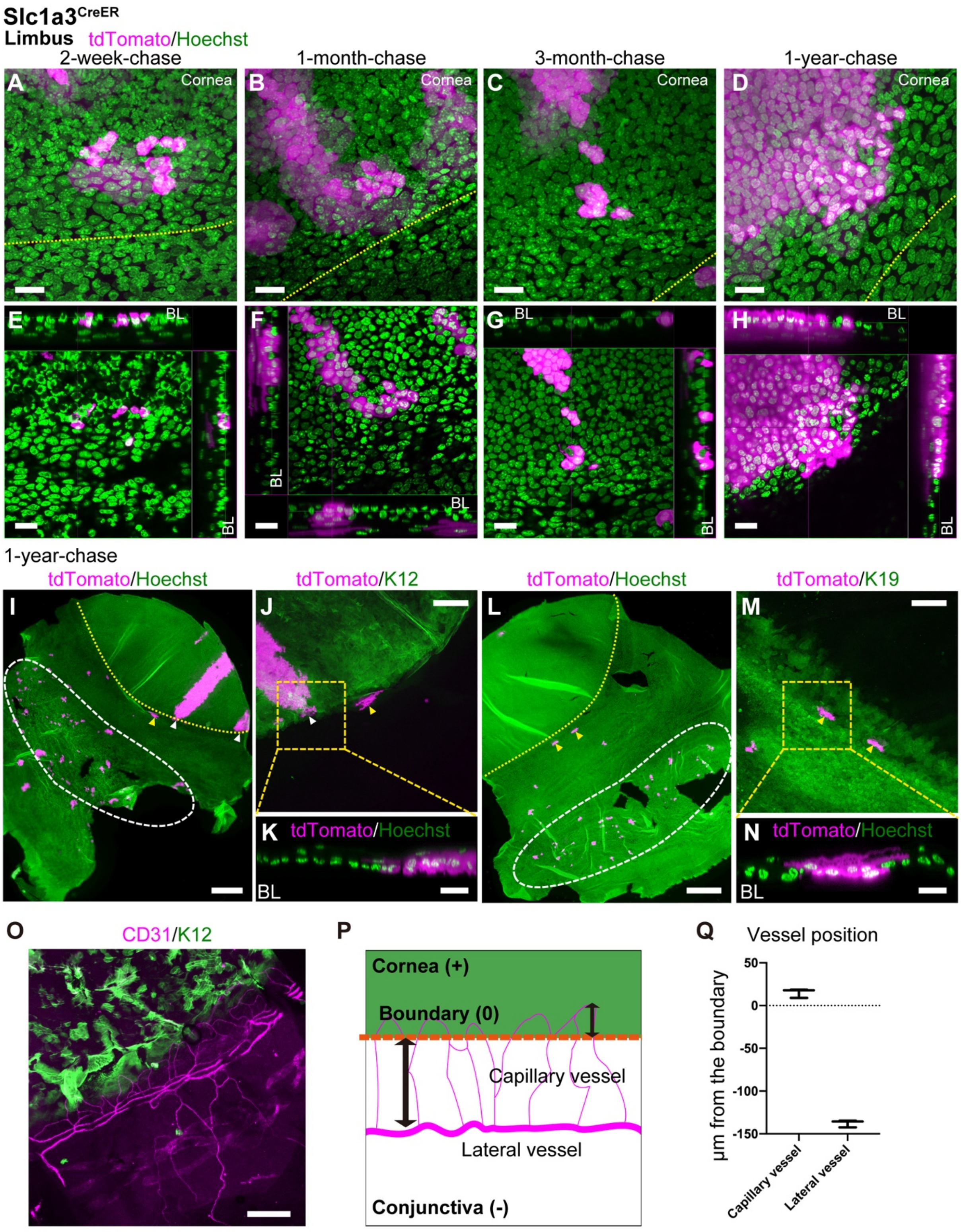
Slc1a3CreER
Limbus tdTomato/Hoechst
2-week-chase
1-month-chase
3-month-chase
1-year-chase
Cornea
BL
1-year-chase
tdTomato/Hoechst
tdTomato/K12
tdTomato/Hoechst
tdTomato/K19
tdTomato/Hoechst
tdTomato/Hoechst
CD31/K12
Cornea (+)
Boundary (0)
Capillary vessel
Lateral vessel
Conjunctiva (-)
Vessel position
µm from the boundary
50
0
-50
-100
-150
Capillary vessel
Lateral vessel

Figure S3. Lineage tracing of Slc1a3^{CreER} in the cornea. (**A-H**) Confocal imaging of representative clones at 2-week-, 1-month-, 3-month-, and 1-year-chases. The areas surrounded by the yellow dashed line in Fig. 2E-H are shown. Images are shown as a maximum-intensity projection (A-D) or confocal sections of the xy, yz, and xz planes of basal clones (E-H; BL, basal layer). The yellow dashed line represents the corneal/conjunctival boundary (A-D). Magenta, tdTomato. Green, Hoechst. Scale bars: 20 µm. (**I-N**) Mouse eyes were analyzed by whole-mount immunostaining at 1-year-chase. The yellow dashed line indicates the corneal/conjunctival boundary. The white dashed line represents the tdTomato+ cell-enriched area in the fornix conjunctiva. Central corneal areas are shown with higher magnification (J, M) and as a side view of Z-stack confocal images (K, N; BL, basal layer). White arrowheads indicate tdTomato+ radial stripes extended from the limbus. Yellow arrowheads indicate tdTomato+ clones expanded laterally within the limbal region. Magenta, tdTomato. Green, K12 (J, corneal marker). Green, K19 (M, conjunctival marker). Green, Hoechst (I, K, L, N). Scale bars: 500 µm (I, L), 200 µm (J, M), 20 µm (K, N). (**O**) Immunostaining of wild-type mouse eye on whole-mounts. Magenta, CD31 (blood vessels). Green, K12 (corneal marker). Scale bars: 200 µm. (**P**) Diagram shows the measurement of the length between the peripheral edge of lateral vessel/capillary vessels and the corneal/conjunctival boundary. (**Q**) The positions of blood vessels are measured from the boundary. N = 3 mice. Data are shown as means ± SD.

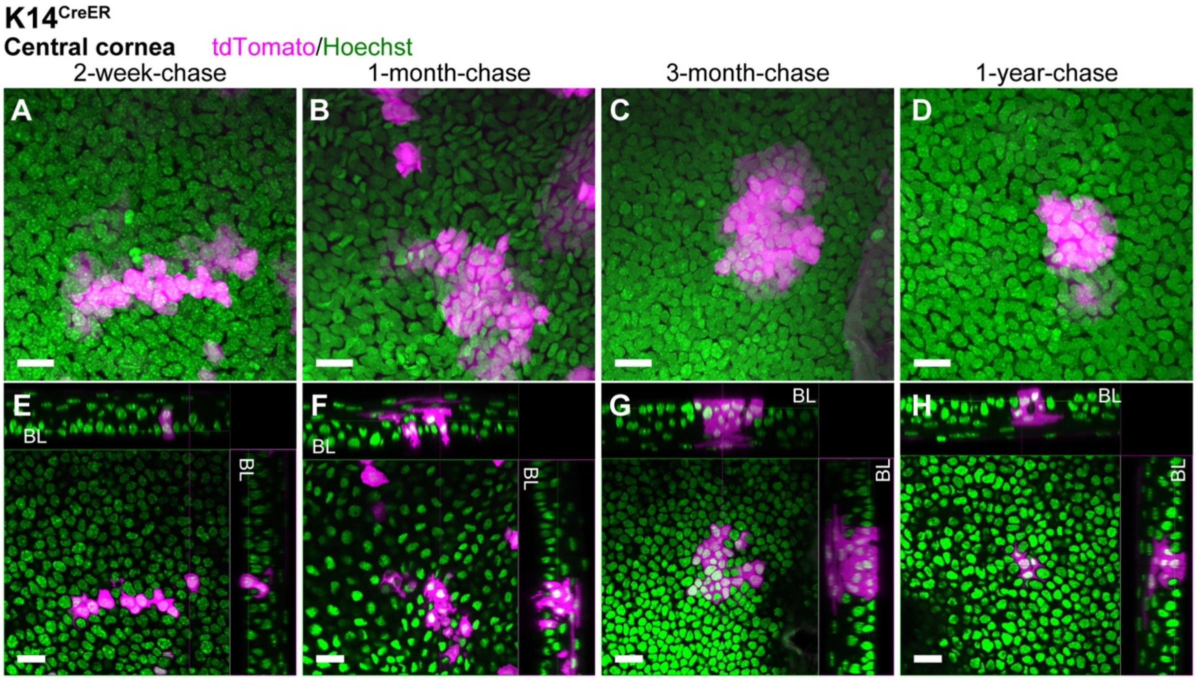

Figure S4. Lineage tracing of K14^{CreER} in the cornea. (**A-H**) Confocal imaging of representative clones at 2-week-, 1-month-, 3-month-, and 1-year-chases. The areas surrounded by the yellow dashed line in Fig. 3E-H are shown. Images are shown as a maximum-intensity projection (A-D) or confocal sections of the xy, yz, and xz planes of basal clones (E-H; BL, basal layer). Magenta, tdTomato. Green, Hoechst. Scale bars: 20 µm.

Development • Supplementary information

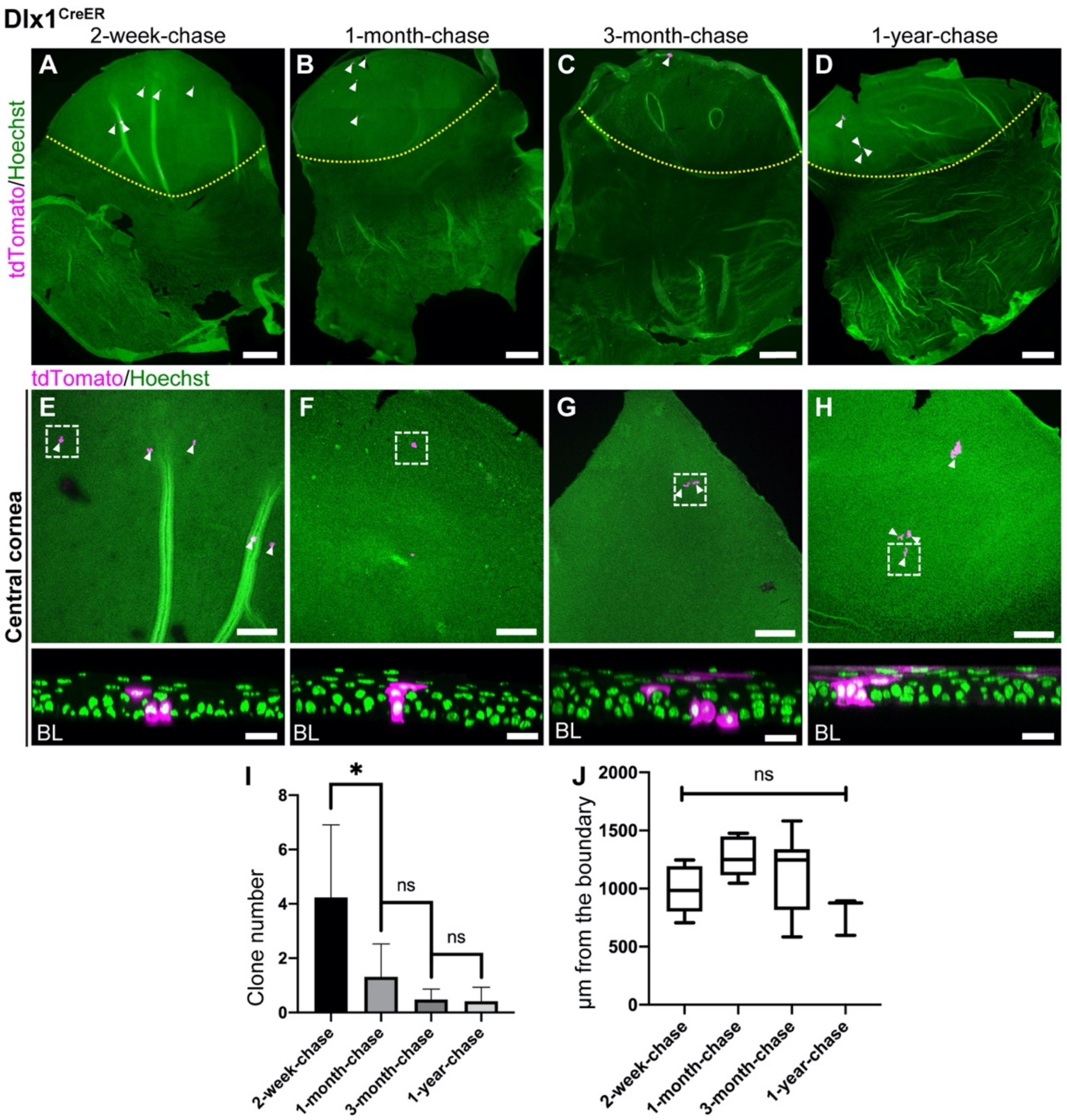

Figure S5. Lineage tracing of Dlx1^{CreER} in the cornea. (**A-H**) Mouse eyes were analyzed by whole-mount immunostaining at 2-week-, 1-month-, 3-month-, and 1-year-chases. Central corneal areas are shown as a maximum-intensity projection (E-H, top). The areas surrounded by white dashed lines are shown as a side view of Z-stack confocal images (E-H, bottom; BL, basal layer). The yellow dashed line indicates the corneal/conjunctival boundary (A-D). Arrowheads indicate tdTomato+ cells (A-H). Magenta, tdTomato. Green, Hoechst. Scale bars: 500 µm (A-D), 200 µm (E-H, top), 20 µm (E-H, bottom). (**I**) The number of tdTomato+ clones per half whole-mount sample is quantified at indicated time points. One-way ANOVA followed by Bonferroni test. *; $P < 0.05$. ns: not significant. (**J**) Box plot showing the distance of tdTomato+ clones from the corneal/conjunctival boundary at indicated time points of chase. $N = 3$ mice at 2-week-chase, $N = 3$ mice at 4-week-chase, $N = 7$ mice at 3-month-chase, and $N = 6$ mice at 1-year-chase. All tdTomato+ clones in whole-mount samples from a half eye were measured and used for quantification. Data are shown as means ± standard deviation (SD). One-way ANOVA followed by Bonferroni test. ns: not significant.

Development • Supplementary information

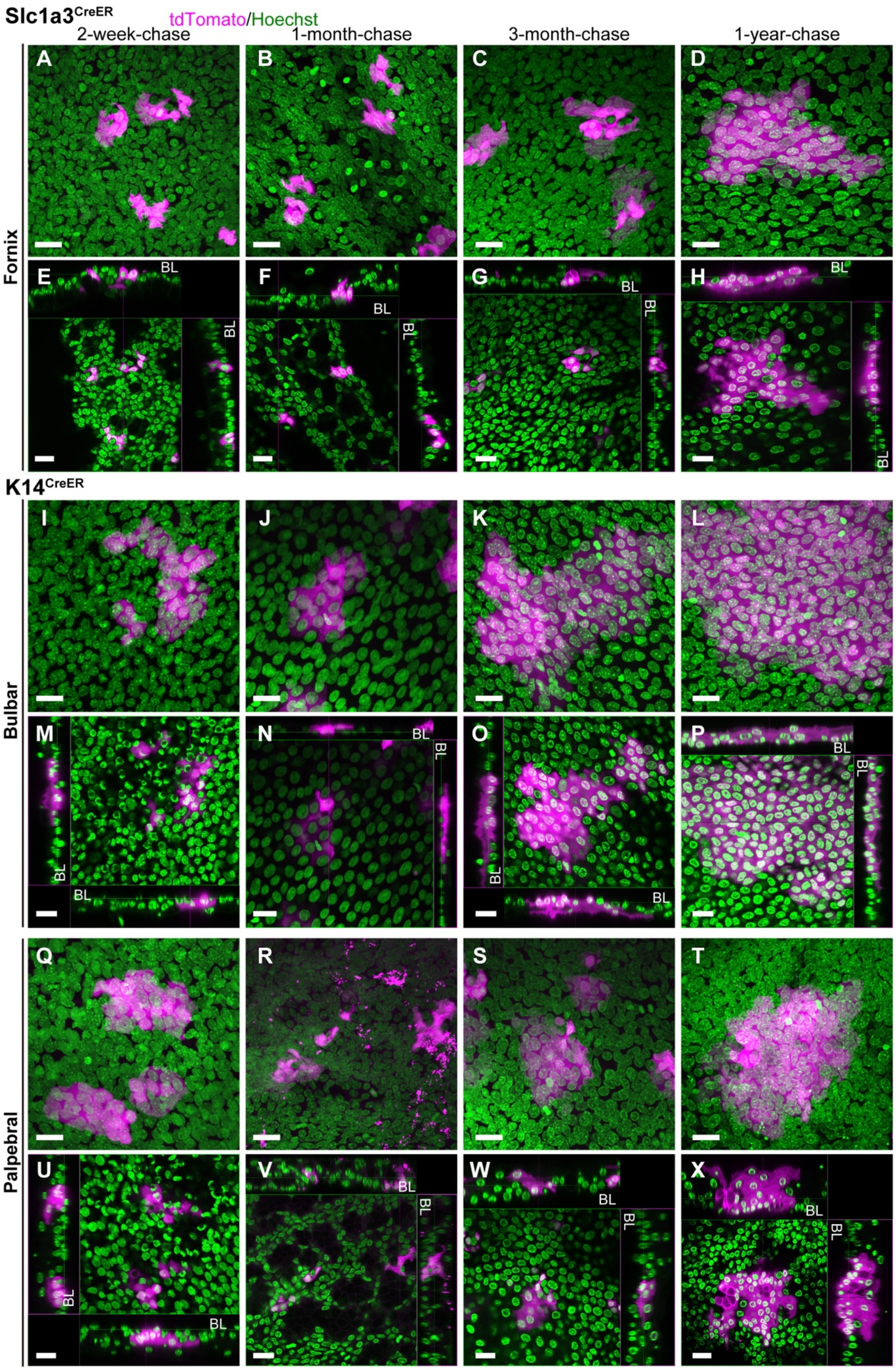
Slc1a3CreER
tdTomato/Hoechst
2-week-chase
1-month-chase
3-month-chase
1-year-chase
Fornix
K14CreER
Bulbar
Palpebral
A
B
C
D
E
F
G
H
I
J
K
L
M
N
O
P
Q
R
S
T
U
V
W
X
BL

Figure S6. Lineage tracing of Slc1a3^{CreER} and K14^{CreER} in the conjunctiva. (**A-H**) Confocal imaging of representative clones of Slc1a3^{CreER} in the fornix conjunctiva at 2-week-, 1-month-, 3-month-, and 1-year-chases. The areas surrounded by the yellow dashed line in Fig. 4A-D are shown. Images are shown as a maximum-intensity projection (A-D) or confocal sections of the xy, yz, and xz planes of basal clones (E-H; BL, basal layer). Magenta, tdTomato. Green, Hoechst. Scale bars: 20 µm. (**I-X**) Confocal imaging of representative clones of K14^{CreER} in the bulbar and palpebral conjunctiva at 2-week-, 1-month-, 3-month-, and 1-year-chases. The areas surrounded by the yellow dashed line in main Fig. 4I-P are shown. Images are shown as a maximum-intensity projection (I-L, Q-T) or confocal sections of xy, yz, and xz planes of basal clones (M-P, U-X; BL, basal layer). Magenta, tdTomato. Green, Hoechst. Scale bars: 20 µm.

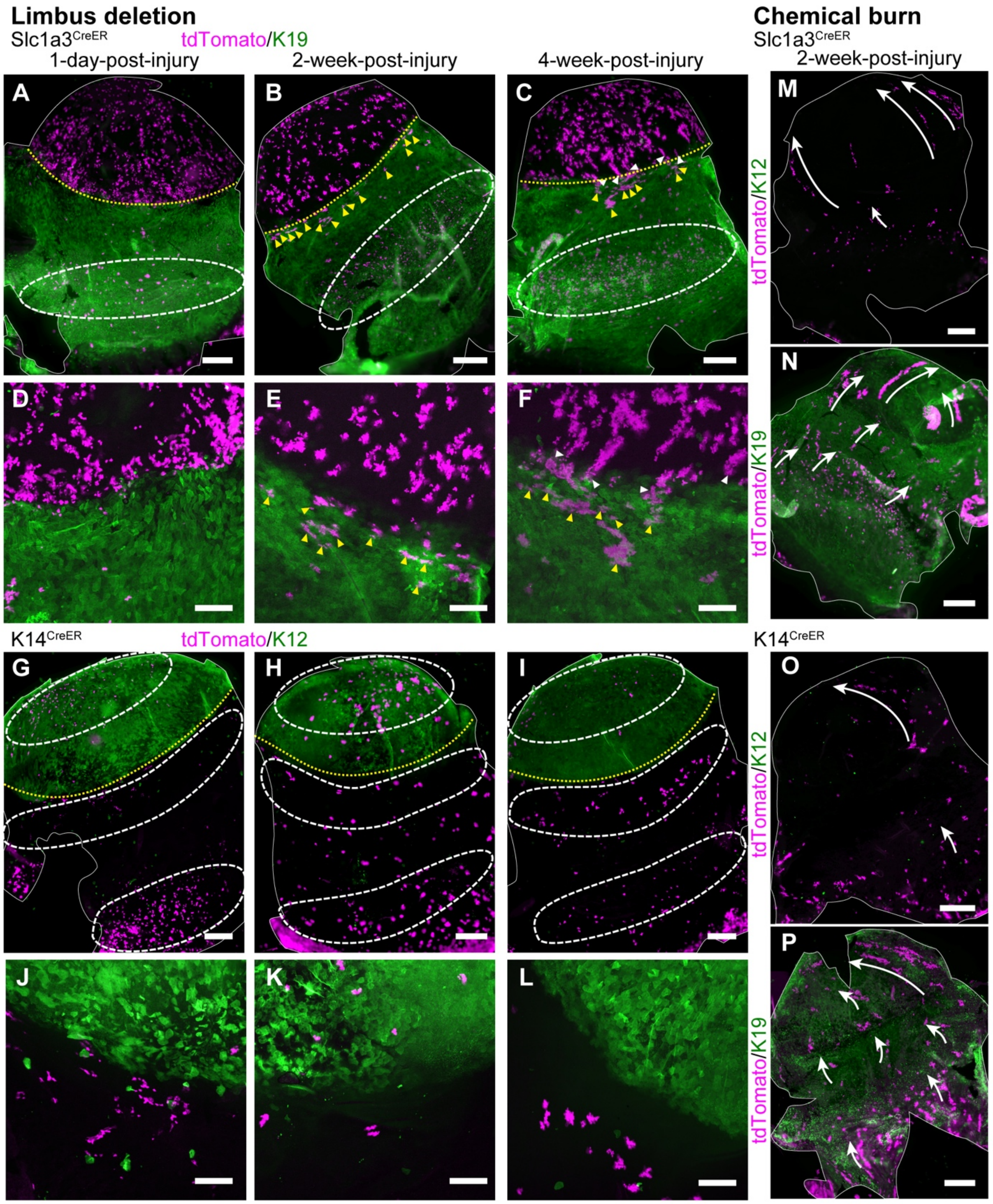
Limbus deletion
Slc1a3CreER
tdTomato/K19
1-day-post-injury
2-week-post-injury
4-week-post-injury
A
B
C
D
E
F
K14CreER
tdTomato/K12
G
H
I
J
K
L
Chemical burn
Slc1a3CreER
2-week-post-injury
M
tdTomato/K12
N
tdTomato/K19
K14CreER
O
tdTomato/K12
P
tdTomato/K19

Figure S7. Time course of injury experiments. (**A-L**) Whole-mount immunostaining after limbal epithelial deletion. Data are shown 1-day-, 2-weeks-, and 4-weeks post-injury. The white line outlines the whole-mount epithelial sheets. The yellow dashed line indicates the corneal/conjunctival boundary. The white dashed line represents the tdTomato+ cell-enriched area. White arrowheads indicate tdTomato+ radial stripes extending from the limbus. Yellow arrowheads indicate tdTomato+ clones expanding laterally within the limbal region. Magenta, tdTomato. Green, K19 (A-F, conjunctival marker) or K12 (G-L, corneal marker). Scale bars: 500 μm (A-C, G-I), 200 μm (D-F, J-L). (**M-P**) Whole-mount immunostaining after chemical burn. Data are shown 2-weeks post-injury. The white line outlines the whole-mount epithelial sheets. White arrows represent the movement of conjunctival tdTomato+ clones. Magenta, tdTomato. Green, K12 (M, O, corneal marker) or K19 (N, P, conjunctival marker). Scale bars: 500 μm.

Development • Supplementary information
