# Supplementary material for: Defining compartmentalized stem cell populations with distinct cell division dynamics in the ocular surface epithelium
Source: Development. 2020 Dec 16;147(24):dev197590. doi: 10.1242/dev.197590 (PMC7758628; doi:10.1242/dev.197590)
Supplement: Reviewer comments [file develop-147-197590_review_history.pdf]

Defining compartmentalized stem cell populations with distinct cell division dynamics in the ocular surface epithelium

Ryutaro Ishii, Hiromi Yanagisawa and Aiko Sada
DOI: 10.1242/dev.197590

Editor: Patrick Tam

Review timeline
Original submission: 6 October 2020
Editorial decision: 8 October 2020
First revision received: 2 November 2020
Accepted: 4 November 2020

Submission to Review Commons

Reviewer 1

Evidence, reproducibility and clarity

In this paper Ishii et al set out to identify distinct progenitor populations in the mouse ocular epithelium using three different transgenic lines. Using EDU pulse chase and label analysis they do in fact identify differences between the three lines and conclude there are distinct populations (Long lived SCs and non LRCs) with unique behaviours. Finally they employ two different damage paradigms (epithelial deletion and burn) to identify potential differences in these cell populations after injury.

The first characterisations is a very nice descriptive piece of work. The figures are beautiful and easy to follow. I feel that their conclusions are justified based on the images and descriptions. There was no statement/justification for the selection of the particular timepoints to start the EDU/CRE induction. Why is this stage selected? I do wonder if the induction of the CRE could be done at more that one time point to potentially identify different populations that may arise over time (also does the ageing epithelium also alter population dynamics?) but this is a minor point outside fo the scope of this study.

The identification of the distinct populations relies entirely on the use of three different mouse transgenic strains. This is very useful but do these genes also specifically label these populations. I.e. The authors should make some effort to identify molecular markers (even in situ/antibody for slc1a3/dlx1/K14 themselves) to label these populations without transgenes. This increases the impact of the study and would further provide molecular information on these cell populations.

Minor Comments

Are prior studies referenced appropriately?

Yes

Are the text and figures clear and accurate?

Overall yes. The introduction to the paper was very nice and laid the groundwork for understanding their study. My only small comments are that it is quite long and the sections describing previous

© 2020. Published by The Company of Biologists under the terms of the Creative Commons Attribution License (https://creativecommons.org/licenses/by/4.0/).

models can be reduced for conciseness. Also it may be helpful to have the English looked over by a native speaker as there are a few sections where it can be improved.

Do you have suggestions that would help the authors improve the presentation of their data and conclusions?

The authors should consider changing their figures to green-magenta for colourblind readers.

Are the data and the methods presented in such a way that they can be reproduced?

Yes

Are the experiments adequately replicated and statistical analysis adequate?

Yes, but exact n numbers should be included in the methods.

Significance

I think this is a very nice characterisation of three transgenic mouse lines where they identify different population dynamics. I feel like minimal work is required for submission before publication in a peer reviewed journal.

My expertise falls outside of this particular field in ocular epithelia so it's challenging for me to give a strong statement on the significance of the identification of these distinct populations. But I do feel this work will be of interest to many in the field. I also think this work would be more impactful if molecular markers were identified to label these populations without needing the transgenic lines so many can benefit from their discovery.

REFEREE'S CROSS-COMMENTING:

I have read the comments from referee 2. I too agree that our reviews are compatible. Labeling and clear explanation of panels in figures will improve the clarity of the manuscript.

Reviewer 2

Evidence, reproducibility and clarity

Objective of study:

Corneal injury is dangerous, since it can initiate stem cell loss, followed by invasion of stem and/or progenitor cells from the conjunctiva. As a consequence, opacity of the cornea and neovascularization will emerge, which eventually can lead to loss of vision. From a medical point of view, a full understanding of the cellular and molecular processes underlying these vision-endangering events is essential.

This MS deals with questions of stem/progenitor cell populations in the ocular surface epithelium of three transgenic mouse lines, their particular localizations within different parts of it (different parts of conjunctiva, limbus, and cornea), and possibilities of their migratory pathways, e.g., towards the corneal center under normal and injured conditions. Or question/topic in brief: Where do stem cells in normal and injured cornea come from? From within center cornea, or do they migrate there from the particular places of the conjunctiva?

Methods used:

Three transgenic CreER mouse lines (Slc1a3 to detect LRCs - "label retaining cells"; K14 and Dlx1 for non-LRCs; see major comment below) are used in EdU pulse chase in vivo experiments (EdU treatment for 1 week, followed by Tamoxifen injection to allow for CreER cell lineage tracing;

© 2020. Published by The Company of Biologists under the terms of the Creative Commons Attribution License (https://creativecommons.org/licenses/by/4.0/).

chase periods are up to 1 year). After completion of chase, mice were euthanized, eyes enucleated, and samples processed for sectioning; alternatively, the ocular epithelium of half an eye was separated from mesenchyme and processed for CreER lineage tracing in whole-mounted epithelial sheets. Different degrees of injury were applied by chemical impact or by mechanical tissue deletion. Migratory distances of cells clones were determined by measurements relative to limbus/cornea borderline.

Results:

In 1st section, distribution of K14, Dlx1 and Slc1 cells (e.g., of non-LRCs and LRCs, respectively) in specific subareas of the ocular epithelium is documented. Parts of these results are confirmatory. In 2nd section, two migratory paths of Slc1a3+ cells were detected, a major radial one with cells migrating towards the cornea center, and a minor lateral pathway along the limbus border. By applying different chase periods and measuring distance of clones from the limbus border, it is indicated that some of the radial clones are faster cycling and shorter-lived, while the lateral ones appeared to divide extremely slowly. In 3rd section, it is documented that many Dlx1+, or K14+ cells are found in central cornea.
They were found still after 3 months chase, but had mostly vanished after 1 year chase, which led the authors to postulate that these clones represented shorter-lived progenitor cells. Then, in 4th section, long-lived LRCs were detected to the fornix region, which appeared to not move away from there. The two markers for non-LRCs were also found in other distinct areas of the conjunctiva (bulbur or palpebral regions). The last section (5th) deals with effects after corneal injury. After limbus tissue deletion, Slc1a3+ cells, e.g., slowly cycling LRCs quickly were activated to replenish limbal tissue. After alkaline burns, Slc1a3+ cells migrated towards the cornea center, while K14+ cells from their particular conjunctival locations moved towards the limbus to possibly compensate for the cell loss there.

Discussion:

The Discussion section mostly is satisfactory. However, relevance of these findings from mice should be discussed as to their possible relevance for human eyes and vision diseases.

Major comments

1. To my frustration: Throughout this study, the K14 and Dlx1 markers are used to mark non-LRCs, while Slc1a3 is interpreted as to label LRCs. However, to my frustrating confusion, in 2nd para of Results this is stated vice versa (p. 6, upper): what´s wrong here? ...is it a typing or other language problem? ...do I get something wrong?
2. Check quantification procedure (statistics)?: I understand that all clones from 1 whole-mounted half eye are used for quantification, and this is done for 3 mice (= 3 half eyes quantification)? The data are combined and averaged? What happened with the second eye from each mouse? These details must be provided in M&M.
3. In Figures, spatial relations between whole-mounted sample and section shown below remains unclear.
4. What do we learn from sections vs. pictures from whole-mounted sheets? Findings from sections remain nearly unnoticed throughout the text; if mentioned, a precise numbering is missing: give precise Fig. No., e.g. "Fig. 3C, left frame".
5. Some interpretations of results are speculative, and do not belong in Results section; e.g., p. 11, lower para: this statement in Results is highly speculative.
6. P. 14, upper, Discussion, 1st para: does this study really provide data on "cell division frequency"? (see also p. 8: type "..suggest the possible heterogeneity of the ocular epithelium regarding its cell division..."): wording "cell division dynamics" might be more appropriate.

Minor points:

- Labels in figures: where is basal, where apical in sections? Label.
- Fig. 4: the many white triangles within pictures are confusing: are these necessary/helpful?
- Fig. 1E, middle: use different line symbols for both features.
- "IFE" in results: what is it? ...explain in text.
- P. 8: type ...behavior of the LRC population.

© 2020. Published by The Company of Biologists under the terms of the Creative Commons Attribution License (https://creativecommons.org/licenses/by/4.0/).

Significance

This study is relevant for basic ocular sciences, and, in particular, for biomedical issues of stem cell-based tissue repair after corneal injuries.

REFEREE'S CROSS-COMMENTING:

I have read the comments of referee 1: I agree with most of his suggestions. I feel that our two reviews of this work are highly compatible.

Author response to reviewers' comments

August 31, 2020

Re: RC-2020-00371

We are thankful to the editor and the reviewers for their insightful comments and suggestions that made us improve the quality of our manuscript.
We have provided our point-by-point responses to the reviewers' comments.

Reviewer #1 (Evidence, reproducibility and clarity (Required)):

In this paper Ishii et al set out to identify distinct progenitor populations in the mouse ocular epithelium using three different transgenic lines. Using EDU pulse chase and label analysis they do in fact identify differences between the three lines and conclude there are distinct populations (Long lived SCs and non LRCs) with unique behaviours. Finally they employ two different damage paradigms (epithelial deletion and burn) to identify potential differences in these cell populations after injury.

The first characterisations is a very nice descriptive piece of work. The figures are beautiful and easy to follow. I feel that their conclusions are justified based on the images and descriptions. There was no statement/justification for the selection of the particular timepoints to start the EDU/CRE induction. Why is this stage selected? I do wonder if the induction of the CRE could be done at more that one time point to potentially identify different populations that may arise over time (also does the ageing epithelium also alter population dynamics?) but this is a minor point outside fo the scope of this study.

We thank the reviewer for the critical comment. All EdU and Tamoxifen treatment was started in mature adult mice at 2- to 4-month-old after intensive postnatal eye growth is ceased and considered to be a steady-state homeostatic condition with minimal effects of aging (Kalha et al., *Stem Cells* 2018). Based on our previous experiments in the skin, induction age (perinatal, young adult, old) didn't affect the labeling pattern. In the revised manuscript, we provided justification of the stage used with a reference by Kalha et al (page 7, lines 14-17, page 17, lines 22-23, page 18, lines 8-9).

The identification of the distinct populations relies entirely on the use of three different mouse transgenic strains. This is very useful but do these genes also specifically label these populations. I.e. The authors should make some effort to identify molecular markers (even in situ/antibody for slc1a3/dlx1/K14 themselves) to label these populations without transgenes. This increases the impact of the study and would further provide molecular information on these cell populations.

As the reviewer suggested, we tried whole-mount and section immunostaining of candidate markers that were previously identified as LRC or non-LRC genes in skin or reported as SC or differentiation

© 2020. Published by The Company of Biologists under the terms of the Creative Commons Attribution License (https://creativecommons.org/licenses/by/4.0/).

markers in the ocular surface epithelium. Among 20 antibodies tested, we found 4 markers (Ifitm3, K14, K36, K13) showed heterogeneous expression patterns in the cornea and conjunctiva (Figure shown below). Ifitm3, which was recently identified as a limbal marker (Altshuler et al., *bioRxiv* 2020), stained preferentially the LRC compartment, i.e. the limbus and fornix conjunctiva. In contrast, K14 showed an enriched staining in the non-LRC compartment (central cornea, bulbar and palpebral conjunctiva) similar to the $K14^{CreER}$ labeling. K36 and K13 were expressed in the central cornea and bulbar/palpebral conjunctiva, respectively, which serve as specialized regional markers of corneal and conjunctival non-LRC compartments. Slc1a3 antibody staining as well as other putative markers (Sox9, MCSP, Sca1, K17, K84, Desmoglein 1, K10, K4, Mt1-2, Collagen IV, Laminin, Tenascin C, YAP, TSP1) didn't work. In the full revision, we will stain these 4 markers (Ifitm3, K14, K36, K13) in EdU pulse-chased mice and

Figure

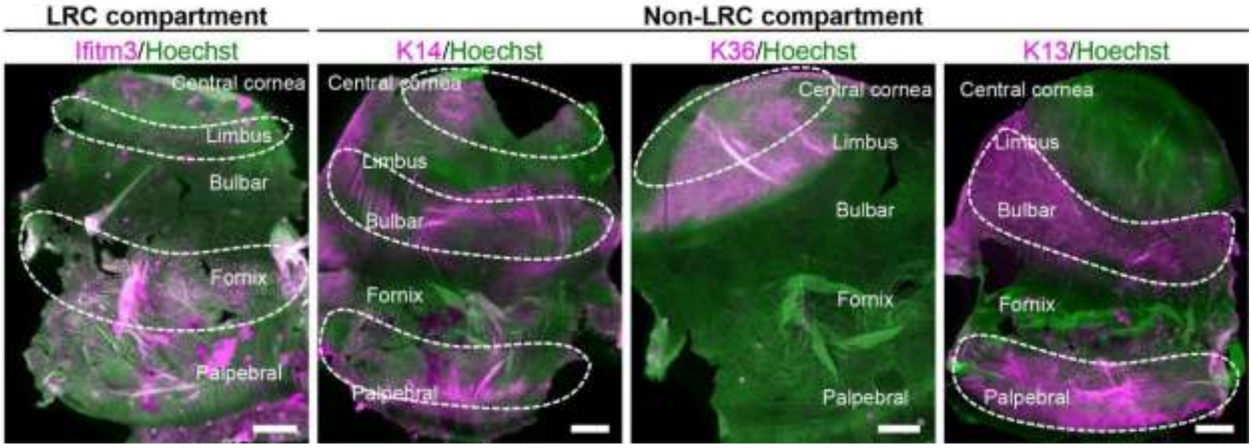

repeat experiments for *N*=3.

Minor Comments

Are prior studies referenced appropriately?

Yes

Are the text and figures clear and accurate?

Overall yes. The introduction to the paper was very nice and laid the groundwork for understanding their study. My only small comments are that it is quite long and the sections describing previous models can be reduced for conciseness. Also it may be helpful to have the English looked over by a native speaker as there are a few sections where it can be improved.

According to the reviewer's comments, we have shortened the introduction in the revised manuscript (page 3 to 7). For the English-language improvement, we will send our manuscript for academic proofreading once text is finalized after a full revision.

Do you have suggestions that would help the authors improve the presentation of their data and conclusions?

The authors should consider changing their figures to green-magenta for colourblind readers.

Thank you for the reviewer's critical comments. We have changed all figures to green-magenda instead of triple colour, blue, green and red.

Are the data and the methods presented in such a way that they can be reproduced?

Yes

© 2020. Published by The Company of Biologists under the terms of the Creative Commons Attribution License (https://creativecommons.org/licenses/by/4.0/).

Are the experiments adequately replicated and statistical analysis adequate?

Yes, but exact n numbers should be included in the methods.

According to the reviewer's comments, we added n numbers in methods and figure legends in the revised manuscript.

Reviewer #1 (Significance (Required)):

I think this is a very nice characterisation of three transgenic mouse lines where they identify different population dynamics. I feel like minimal work is required for submission before publication in a peer reviewed journal.

My expertise falls outside of this particular field in ocular epithelia so it's challenging for me to give a strong statement on the significance of the identification of these distinct populations. But I do feel this work will be of interest to many in the field. I also think this work would be more impactful if molecular markers were identified to label these populations without needing the transgenic lines so many can benefit from their discovery.

We appreciate the reviewer's positive comments and suggestions. As per reviewer's suggestions, we performed additional staining experiments and identified putative markers for LRC and non- LRC compartments (Ifitm3, K14, K36, K13), which is crucial to support the main conclusions of our paper. After a full revision, our new data will provide useful tools to define distinct stem cell population in the ocular surface epithelium, which has been missing in the field.

REFEREE'S CROSS-COMMENTING:

I have read the comments from referee 2. I too agree that our reviews are compatible. Labeling and clear explanation of panels in figures will improve the clarity of the manuscript.

Reviewer #2 (Evidence, reproducibility and clarity (Required)):

Objective of study:

Corneal injury is dangerous, since it can initiate stem cell loss, followed by invasion of stem and/or progenitor cells from the conjunctiva. As a consequence, opacity of the cornea and neovascularization will emerge, which eventually can lead to loss of vision. From a medical point of view, a full understanding of the cellular and molecular processes underlying these vision-endangering events is essential.

This MS deals with questions of stem/progenitor cell populations in the ocular surface epithelium of three transgenic mouse lines, their particular localizations within different parts of it (different parts of conjunctiva, limbus, and cornea), and possibilities of their migratory pathways, e.g., towards the corneal center under normal and injured conditions. Or question/topic in brief: Where do stem cells in normal and injured cornea come from? From within center cornea, or do they migrate there from the particular places of the conjunctiva?

Methods used:

Three transgenic CreER mouse lines (Slc1a3 to detect LRCs - "label retaining cells"; K14 and Dlx1 for non-LRCs; see major comment below) are used in EdU pulse chase in vivo experiments (EdU treatment for 1 week, followed by Tamoxifen injection to allow for CreER cell lineage tracing; chase periods are up to 1 year). After completion of chase, mice were euthanized, eyes enucleated, and samples processed for sectioning; alternatively, the ocular epithelium of half an eye was separated from mesenchyme and processed for CreER lineage tracing in whole-mounted epithelial sheets. Different degrees of injury were applied by chemical impact or by mechanical tissue

© 2020. Published by The Company of Biologists under the terms of the Creative Commons Attribution License (https://creativecommons.org/licenses/by/4.0/).

deletion. Migratory distances of cells clones were determined by measurements relative to limbus/cornea borderline.

Results:

In 1st section, distribution of K14, Dlx1 and Slc1 cells (e.g., of non-LRCs and LRCs, respectively) in specific subareas of the ocular epithelium is documented. Parts of these results are confirmatory. In 2nd section, two migratory paths of Slc1a3+ cells were detected, a major radial one with cells migrating towards the cornea center, and a minor lateral pathway along the limbus border. By applying different chase periods and measuring distance of clones from the limbus border, it is indicated that some of the radial clones are faster cycling and shorter-lived, while the lateral ones appeared to divide extremely slowly. In 3rd section, it is documented that many Dlx1+, or K14+ cells are found in central cornea. They were found still after 3 months chase, but had mostly vanished after 1 year chase, which led the authors to postulate that these clones represented shorter-lived progenitor cells. Then, in 4th section, long-lived LRCs were detected to the fornix region, which appeared to not move away from there. The two markers for non-LRCs were also found in other distinct areas of the conjunctiva (bulbur or palpebral regions). The last section (5th) deals with effects after corneal injury. After limbus tissue deletion, Slc1a3+ cells, e.g., slowly cycling LRCs quickly were activated to replenish limbal tissue. After alkaline burns, Slc1a3+ cells migrated towards the cornea center, while K14+ cells from their particular conjunctival locations moved towards the limbus to possibly compensate for the cell loss there.

Discussion:

The Discussion section mostly is satisfactory. However, relevance of these findings from mice should be discussed as to their possible relevance for human eyes and vision diseases.

We appreciate the reviewer's insightful comments. Our study proposed possible heterogeneity within the limbus and identified the previously uncharacterized Slc1a3^{CreER+} slow-cycling, latelally-expanding SC population. This limbal population is inactive during normal homeostasis, but it rapidly expands in response to injury to regenerate ocular surface epithelium, particularly useful after extensive ocular damages such as Stevens-Johnson syndrome or severe chemical burns. Our genetic tools will contribute to further address SC dynamics in different disease or injury models in vivo and to find potential therapeutic strategy for limbal SC deficiency. The epithelial SC heterogeneity may also be relevant for understanding the mechanisms of tumorigenesis and for in vitro reconstitution of 3D eye tissues in future. In the revised manuscript, we added discussion about possible relevance of observed SC heterogeneity for clinical application in future (page 16, lines 14-17 and page 17, lines 1-9).

Major comments

1. To my frustration: Throughout this study, the K14 and Dlx1 markers are used to mark non- LRCs, while Slc1a3 is interpreted as to label LRCs. However, to my frustrating confusion, in 2nd para of Results this is stated vice versa (p. 6, upper): what´s wrong here? ...is it a typing or other language problem? ...do I get something wrong?

We are thankful for pointing this out. In our previous study in the mouse skin epidermis, Dlx1 and Slc1a3 markers were identified as LRCs and non-LRCs (stated in p. 6, upper in the original manuscript). In contrast, Dlx1 and Slc1a3 markers showed an opposite pattern in the ocular surface epithelium (Dlx1 in non-LRCs and Slc1a3 in LRCs). To avoid any confusion, we have carefully changed some description in the revised manuscript (page 8, lines 7-8 and page 8, lines 16-19).

2. Check quantification procedure (statistics)?: I understand that all clones from 1 whole-mounted half eye are used for quantification, and this is done for 3 mice (= 3 half eyes quantification)? The data are combined and averaged? What happened with the second eye from each mouse? These details must be provided in M&M.

© 2020. Published by The Company of Biologists under the terms of the Creative Commons Attribution License (https://creativecommons.org/licenses/by/4.0/).

We are thankful to the reviwer's suggestion to clarify the quantification methods. We counted all clones from a half eye per mouse and repeated experiments with N=3 mice for Slc1a3^{CreER} and K14^{CreER}. Due to low efficiency of Cre, we used N=3 to 7 mice for Dlx1^{CreER}. N numbers are shown in figure legends and methods in the revised manuscript. Data are shown as the average of an individual mouse with standard deviation (SD). We used 1 eye for K12 and 1 eye for K19 staining.

3. In Figures, spatial relations between whole-mounted sample and section shown below remains unclear.

Thank you for the reviewer's suggestion. In the revised figures, we added a yellow dotted square in the whole-mounted image to indicate the area shown in the section view (new Figure2e-h, Figure3e-h, Figure4a-d, Figure4i-p). We also added the original Z-stack images for projection and ortho (section) view in supplemental figures to indicate the relationship between whole- mounted sample and section view (new FigureS2a-h, FigureS3, FigureS5).

4. What do we learn from sections vs. pictures from whole-mounted sheets? Findings from sections remain nearly unnoticed throughout the text; if mentioned, a precise numbering is missing: give precise Fig. No., e.g. "Fig. 3C, left frame".

As per reviewer's suggestion, we renumbered each figure and refered individual panel in the text. We also included some descriptions in the revised manuscript to explain the interpretation of section results.

5. Some interpretations of results are speculative, and do not belong in Results section; e.g., p. 11, lower para: this statement in Results is highly speculative.

We agree with the reviewer that our interpretation of the relationship between K14^{CreER}- and Slc1a3^{CreER}-derived clones was speculative (on p. 11, lower paragraph). In the revised manuscript, we removed the description.

6. P. 14, upper, Discussion, 1st para: does this study really provide data on "cell division frequency"? (see also p. 8: type "..suggest the possible heterogeneity of the ocular epithelium regarding its cell division..."): wording "cell division dynamics" might be more appropriate.

We agree with the reviewer's suggestion. We have changed the word "cell division frequency" to "cell division dynamics" in the title and text.

Minor points:

- Labels in figures: where is basal, where apical in sections? Label.

Thank you for the reviewer's comment. All z-stack images were arranged as the basal on the bottom and the apical on the top. We labeled all section images with "BL" to indicate basal layer in the revised manuscript.

- Fig. 4: the many white triangles within pictures are confusing: are these necessary/helpful?

We agree with the reviewer's comment. We deleted all white arroheads from Figure 4.

- Fig. 1E, middle: use different line symbols for both features.

We have changed the colour of each line symbol.

- "IFE" in results: what is it? ...explain in text.

Thank you for the reviewer's comment. IFE is the abbreviation of interfollicle epidermis of skin. Since this abbreviation is not commmon, we spelled out as interfollicle epidermis in the revised manuscript.

© 2020. Published by The Company of Biologists under the terms of the Creative Commons Attribution License (https://creativecommons.org/licenses/by/4.0/).

- P. 8: type ...behavior of the LRC population...

We revised the manuscript accordingly.

Reviewer #2 (Significance (Required)):

This study is relevant for basic ocular sciences, and, in particular, for biomedical issues of stem cell-based tissue repair after corneal injuries.

REFEREE'S CROSS-COMMENTING:

I have read the comments of referee 1: I agree with most of his suggestions. I feel that our two reviews of this work are highly compatible.

Submission to Development

First decision letter

MS ID#: DEVELOP/2020/197590

MS TITLE: Defining compartmentalized stem and progenitor populations with distinct cell division dynamics in the ocular surface epithelium

AUTHORS: Ryutaro Ishii, Hiromi Yanagisawa, and Aiko Sada

Thanks for transfer the manuscript from Review Common to Development. I have evaluated the your response to the review and the revision of the manuscript and recognised the merit of your work. We are prepared to consider publishing this work if the issue outlined in the Editor's Note (appended to this letter) can be addressed satisfactorily.

Please also attend to the comments in the previous review and ensure that you clearly highlight all changes made in the revised manuscript. Please avoid using 'Tracked changes' in Word files as these are lost in PDF conversion. I should be grateful if you would also provide a point-by-point response detailing how you have dealt with the points raised by the reviewers in the 'Response to Reviewers' box. If you do not agree with any of their criticisms or suggestions please explain clearly why this is so. Please also note that Development will normally permit only one round of revision.

We are aware that you may be experiencing disruption to the normal running of your lab that make experimental revisions challenging. If it would be helpful, we encourage you to contact us to discuss your revision in greater detail. Please send us a point-by-point response indicating where you are able to address concerns raised (either experimentally or by changes to the text) and where you will not be able to do so within the normal timeframe of a revision. We will then provide further guidance. Please also note that we are happy to extend revision timeframes as necessary.

Editor's Note

Issues to address:

Inference about contribution to the radial versus lateral clones at different experimental settings: How could the radial and lateral clones be identified as “independent” clones other than that in specimens examined at certain time (e.g. 3 month and I year: Figure 2) that they appeared to be spatially separated. Could they be descendant of the same clone, despite of lack of physical congruence? In this regard, a time-course tracking of clonal expansion would be informative.

© 2020. Published by The Company of Biologists under the terms of the Creative Commons Attribution License (https://creativecommons.org/licenses/by/4.0/).

Figure 2i-g:Did the quantified data on clonal spread (length from the boundary) exclude the laterally expanding clones (see Suppl Fig S1k, S6b, c, e, f)? Given the apparent difference in the contribution to the ocular epithelium by the radial and lateral clones (at 1-year time point - panel h), it would be informative to characterize their pattern of clonal distribution in more detail. These data are essential to support the claim of “a rapid increase of laterally expanding clone” in the response of the SC/progenitors to homeostasis versus repair of injury.
Is there any information on the factors that drive the distinctive clonal behaviour in the context of radial versus lateral expansion?

What may be learned from the response of the SCs to different injury: deletion of the limbal epithelium which spared some Slc1 SCs (which initiate lateral expansion followed by radial expansion) versus the chemical injury which may eliminate all limbal SCs resulting in “conjunctivalization”? May this reveal functional cross-interaction between SCs types in the ocular epithelium in the generation of corneal and conjunctival epithelium at homeostasis?

Specific point:

“infrequently-dividing or “slow-cycling” cells in the bulge region are identified…”: bulge region of what structure?
What is meant by “an equipotent, single-cell population”, “tear film homeostasis through goblet cells?

“multiple conjunctival SCs lost their identity and territorial distribution pattern”; might be appropriate to attribute this behaviour as “ocular epithelial SCs change their differentiation propensity and tissue coverage”.

Figure and Figure legends

Figure 1a, b: suggest revising: …or whole mount preparation of one half of the epithelium of cornea and conjunctiva (blue dotted line).
Figure 1i, j: Consider reducing the use of white arrowheads to mark the cells of interest to allow the reader to evaluate the imaging result of tdTomato+ cells
Figure 5c-j: Where is “the white line outlines the whole mount epithelial sheets” for panel c-j?
Figure 5m: How was the degree of horizontal expansion of limbal clones (e.g. Figure 5d, h, Suppl figure 2k, n) assessed between homeostasis and limbal deletion?
Suppl Figure S2e-h, S3e-h, S5: Which area was marked/surrounded by “yellow dashed line”, or is that the whole frame?What is the rationale for switching the orientation of the apical-basal dimension in different panels of the figure, e.g. Suppl figure S3 e, f vs g, h; Suppl figure S5 e, h vs f, g?
Statistical results: To be included in Fig 2 i-l, 3 i-l, 4 e-h, 4 q-t. Fig S4 i, j

---

Author response to reviewers' comments

November 1, 2020

Re: DEVELOP/2020/197590

We are thankful to the editor and the reviewers for their insightful comments and suggestions that made us improve the quality of our manuscript. We have performed additional experiments and revised our manuscript. We have changed the manuscript format according to the Journal guideline.

The following are our point-by-point responses to the editor and reviewer’s comments.

© 2020. Published by The Company of Biologists under the terms of the Creative Commons Attribution License (https://creativecommons.org/licenses/by/4.0/).

Editor's Note

Issues to address:

Inference about contribution to the radial versus lateral clones at different experimental settings: How could the radial and lateral clones be identified as "independent" clones other than that in specimens examined at certain time (e.g. 3 month and I year: Figure 2) that they appeared to be spatially separated. Could they be descendant of the same clone, despite of lack of physical congruence? In this regard, a time-course tracking of clonal expansion would be informative.

We thank the editor for the critical comments. As pointed out by the editor, these clones were spatially separated (new Fig. 2M, N), but our experiments cannot fully address whether radial and lateral clones are truly "independent". We agree with the editor that the time-course tracking of clonal expansion (e.g., intravital imaging of same clones over 1 year) is very interesting and powerful approach; however, considering the time and nature of the experiment, we feel that it is beyond the scope of the current paper.

In the revised manuscript, we removed the description of "independent" from the results (page 8, lines 207-219) and discussed that these clones could be generated from the same population (page 12, lines 340-343).

Figure 2i-g:Did the quantified data on clonal spread (length from the boundary) exclude the laterally expanding clones (see Suppl Fig S1k, S6b, c, e, f)? Given the apparent difference in the contribution to the ocular epithelium by the radial and lateral clones (at 1-year time point - panel h), it would be informative to characterize their pattern of clonal distribution in more detail. These data are essential to support the claim of "a rapid increase of laterally expanding clone" in the response of the SC/progenitors to homeostasis versus repair of injury.

We thank the editor to raise this important point. In the revised manuscript, we quantified the position of the radial and lateral clones (new Fig. 2M, N) and length of lateral-expanding clones in homeostasis (new Fig. 2O) and after injury (new Fig. 5K, L). These data indicate that lateral clones gradually expanded over 1 year of chase during homeostasis, whereas physical injury induced rapid expansion of these clones at 1 week post injury. We also quantitatively showed that radial and lateral clones were located at distinct positions.

To respond to the editor's first question, we re-plotted original clonal distribution graphs (new Fig. 2I-L, Fig. 3I-K, Fig. 4E-H, Fig. 4Q-T) and included the laterally expanding clones in Fig. 2I-L, which are located within 50 µm of the K12-negative area. For the clones in the conjunctiva, we quantified them starting at 50 µm from the boundary (new Fig. 3I-K, Fig. 4E-H, Fig. 4Q-T). To avoid any confusion, we define the K12 +/- boundary as zero, cornea as positive- and conjunctiva as negative values and revised our graphs (new Fig. 2I-L, Fig. 3I-K, Fig. 4E-H, Fig. 4Q-T) and cartoon accordingly (new Fig. S1K).

Is there any information on the factors that drive the distinctive clonal behaviour in the context of radial versus lateral expansion?

To address the possible involvement of environmental factors, we stained with CD31, a blood vessel marker, and found that the region with lateral clones was enriched with smaller capillary vessels, whereas the region with radial clones was generally avascular (new Fig. S3O-Q). Since capillary vessels deliver oxygen and nutrients, they may create a specialized environment for lateral clones.

What may be learned from the response of the SCs to different injury: deletion of the limbal epithelium which spared some Slc1 SCs (which initiate lateral expansion followed by radial expansion) versus the chemical injury which may eliminate all limbal SCs resulting in "conjunctivalization"? May this reveal functional cross-interaction between SCs types in the ocular epithelium in the generation of corneal and conjunctival epithelium at homeostasis?

We appreciate the editor's insightful comments. This is an interesting interpretation of our data, and we have added it to the discussion (page 13, lines 380-384).

© 2020. Published by The Company of Biologists under the terms of the Creative Commons Attribution License (https://creativecommons.org/licenses/by/4.0/).

Specific point:
"infrequently-dividing or "slow-cycling" cells in the bulge region are identified…": bulge region of what structure?

We appreciate the editor for the careful reading and kind feedback on our manuscript. We added bulge region of "skin hair follicles" to revised manuscript (page 3, line 53).

What is meant by "an equipotent, single-cell population", "tear film homeostasis through goblet cells?

We agree that these descriptions are confusing. We have rephrased to"a homogenous population" (page 3, lines 66-68), and"provides mucins required for the maintenance of the tear film", respectively in the revised manuscript (page 3, lines 74-75).

"multiple conjunctival SCs lost their identity and territorial distribution pattern"; might be appropriate to attribute this behaviour as "ocular epithelial SCs change their differentiation propensity and tissue coverage".

We revised the manuscript accordingly.

Figure and Figure legends
Figure 1a, b: suggest revising: …or whole mount preparation of one half of the epithelium of cornea and conjunctiva (blue dotted line).

We think our previous cartoon was confusing. We revised the cartoon to clearly indicate how section and whole-mount samples are taken (new Fig. 1B).

Figure 1i, j: Consider reducing the use of white arrowheads to mark the cells of interest to allow the reader to evaluate the imaging result of tdTomato+ cells

As per editor's suggestion, we have removed white arrowheads from the Figure (new Fig. 1I, J).

Figure 5c-j: Where is "the white line outlines the whole mount epithelial sheets" for panel c-j?

The white line outline were not included in panel g-j. We revised the figure legends (new Fig. 5C-F).

Figure 5m: How was the degree of horizontal expansion of limbal clones (e.g. Figure 5d, h, Suppl figure 2k, n) assessed between homeostasis and limbal deletion?

We have provided the quantification of the length of lateral-expanding clones in homeostasis (new Fig. 2O) and after injury (new Fig. 5K, L). We showed that the clones were expanded about double in size in both homeostasis and injury.

Suppl Figure S2e-h, S3e-h, S5: Which area was marked/surrounded by "yellow dashed line", or is that the whole frame?

The"yellow dashed line" in lower magnification images (Fig. 2E-H, Fig. 3E-H, Fig. S5E-H) are enlarged and shown in Fig. S3A-H, S4A-H, S5E-H. We revised the figure legends to indicate where yellow dashed lines are from.

What is the rationale for switching the orientation of the apical-basal dimension in different panels of the figure, e.g. Suppl figure S3 e, f vs g, h; Suppl figure S5 e, h vs f, g?

We understand the editor's point. Due to technical difficulty, epithelial sheets could not be distinguished surface vs basal sides after whole-mount staining. Because of this issue, samples were either imaged from basal or surface side on confocal microscope, and the orientation of the apical-basal dimension was not consistent between different panels of the figures.

© 2020. Published by The Company of Biologists under the terms of the Creative Commons Attribution License (https://creativecommons.org/licenses/by/4.0/).

Statistical results: To be included in Fig 2 i-l, 3 i-l, 4 e-h, 4 q-t. Fig S4 i, j

We have provided statistical analysis on new Fig. 3L, Fig. S5I, J and showed how clone number has been changed overtime. The clone position has been shown as a frequency distributions, which explains tendency and pattern of all clones counted (new Fig. 2I-L, 3I-K, 4E-H, 4Q-T).
Since this data is not a comparison between groups, we couldn't obtain any meaningful statistical values.

Reviewer #1 (Evidence, reproducibility and clarity (Required)):
In this paper Ishii et al set out to identify distinct progenitor populations in the mouse ocular epithelium using three different transgenic lines. Using EDU pulse chase and label analysis they do in fact identify differences between the three lines and conclude there are distinct populations (Long lived SCs and non LRCs) with unique behaviours. Finally they employ two different damage paradigms (epithelial deletion and burn) to identify potential differences in these cell populations after injury.

The first characterisations is a very nice descriptive piece of work. The figures are beautiful and easy to follow. I feel that their conclusions are justified based on the images and descriptions. There was no statement/justification for the selection of the particular timepoints to start the EDU/CRE induction. Why is this stage selected? I do wonder if the induction of the CRE could be done at more that one time point to potentially identify different populations that may arise over time (also does the ageing epithelium also alter population dynamics?) but this is a minor point outside fo the scope of this study.

We thank the reviewer for the critical comment. All EdU and Tamoxifen treatment was started in mature adult mice at 2- to 4-month-old after intensive postnatal eye growth is ceased and considered to be a steady-state homeostatic condition with minimal effects of aging (Kalha et al., *Stem Cells* 2018). Based on our previous experiments in the skin, induction age (perinatal, young adult, old) didn't affect the labeling pattern. In the revised manuscript, we provided justification of the stage used with a reference by Kalha et al (page 6, lines 151-154, page 14, lines 407-409, page 15, lines 420-421).

The identification of the distinct populations relies entirely on the use of three different mouse transgenic strains. This is very useful but do these genes also specifically label these populations. I.e. The authors should make some effort to identify molecular markers (even in situ/antibody for slc1a3/dlx1/K14 themselves) to label these populations without transgenes. This increases the impact of the study and would further provide molecular information on these cell populations.

As the reviewer suggested, we tried whole-mount and section immunostaining of candidate markers that were previously identified as LRC or non-LRC genes in skin or reported as SC or differentiation markers in the ocular surface epithelium. Among 20 antibodies tested, we found 3 markers (Ifitm3, K14 and K13) showed heterogeneous expression patterns in the cornea and conjunctiva (new Fig. S2). Ifitm3, which has recently been identified as a limbal marker (Altshuler et al., *bioRxiv* 2020), stained the LRC compartment, i.e. the limbus and fornix conjunctiva, preferentially. In contrast, K14 showed an enriched staining in the non-LRC compartment (central cornea, bulbar and palpebral conjunctiva) similar to the $K14^{CreER}$ labeling. K13 were preferentially expressed in the bulbar/palpebral conjunctiva, which serve as specialized regional markers of conjunctival non-LRC compartment. Slc1a3 antibody as well as other putative markers (Sox9, MCSP, Sca1, K17, K84, Desmoglein 1, K10, K4, Mt1-2, Collagen IV, Laminin, Tenascin C, YAP, TSP1) didn't work. In the revised manuscript, we provided staining of these 3 markers (Ifitm3, K14 and K13) in new Fig. S2.

Minor Comments

Are prior studies referenced appropriately?
Yes

Are the text and figures clear and accurate?
Overall yes. The introduction to the paper was very nice and laid the groundwork for understanding their study. My only small comments are that it is quite long and the sections describing previous

© 2020. Published by The Company of Biologists under the terms of the Creative Commons Attribution License (https://creativecommons.org/licenses/by/4.0/).

models can be reduced for conciseness. Also it may be helpful to have the English looked over by a native speaker as there are a few sections where it can be improved.

According to the reviewer's comments, we have shortened the introduction in the revised manuscript (page 3 to 5). For the English-language improvement, we sent our manuscript for academic proofreading and carefully corrected English errors and writing style in the revised manuscript.

Do you have suggestions that would help the authors improve the presentation of their data and conclusions?

The authors should consider changing their figures to green-magenta for colourblind readers.

Thank you for the reviewer's critical comments. We have changed all figures to green-magenta.

Are the data and the methods presented in such a way that they can be reproduced?
Yes

Are the experiments adequately replicated and statistical analysis adequate?

Yes, but exact n numbers should be included in the methods.

According to the reviewer's comments, we added n numbers in methods and figure legends in the revised manuscript.

Reviewer #1 (Significance (Required)):
I think this is a very nice characterisation of three transgenic mouse lines where they identify different population dynamics. I feel like minimal work is required for submission before publication in a peer reviewed journal.

My expertise falls outside of this particular field in ocular epithelia so it's challenging for me to give a strong statement on the significance of the identification of these distinct populations. But I do feel this work will be of interest to many in the field. I also think this work would be more impactful if molecular markers were identified to label these populations without needing the transgenic lines so many can benefit from their discovery.

We appreciate the reviewer's positive comments and suggestions. As per reviewer's suggestions, we performed additional staining experiments and identified putative markers for LRC and non-LRC compartments (Ifitm3, K14 and K13), which is crucial to support the main conclusions of our paper. Our new data provided useful tools to define distinct stem cell population in the ocular surface epithelium, which has been missing in the field.

REFEREE'S CROSS-COMMENTING:

I have read the comments from referee 2. I too agree that our reviews are compatible. Labeling and clear explanation of panels in figures will improve the clarity of the manuscript.

Reviewer #2 (Evidence, reproducibility and clarity (Required)):

Objective of study:

Corneal injury is dangerous, since it can initiate stem cell loss, followed by invasion of stem and/or progenitor cells from the conjunctiva. As a consequence, opacity of the cornea and neovascularization will emerge, which eventually can lead to loss of vision. From a medical point of view, a full understanding of the cellular and molecular processes underlying these vision-endangering events is essential.

This MS deals with questions of stem/progenitor cell populations in the ocular surface epithelium of three transgenic mouse lines, their particular localizations within different parts of it (different

© 2020. Published by The Company of Biologists under the terms of the Creative Commons Attribution License (https://creativecommons.org/licenses/by/4.0/).

parts of conjunctiva, limbus, and cornea), and possibilities of their migratory pathways, e.g., towards the corneal center under normal and injured conditions. Or question/topic in brief: Where do stem cells in normal and injured cornea come from? From within center cornea, or do they migrate there from the particular places of the conjunctiva?

Methods used:

Three transgenic CreER mouse lines (Slc1a3 to detect LRCs - "label retaining cells"; K14 and Dlx1 for non-LRCs; see major comment below) are used in EdU pulse chase in vivo experiments (EdU treatment for 1 week, followed by Tamoxifen injection to allow for CreER cell lineage tracing; chase periods are up to 1 year). After completion of chase, mice were euthanized, eyes enucleated, and samples processed for sectioning; alternatively, the ocular epithelium of half an eye was separated from mesenchyme and processed for CreER lineage tracing in whole-mounted epithelial sheets. Different degrees of injury were applied by chemical impact or by mechanical tissue deletion. Migratory distances of cells clones were determined by measurements relative to limbus/cornea borderline.

Results:
In 1st section, distribution of K14, Dlx1 and Slc1 cells (e.g., of non-LRCs and LRCs, respectively) in specific subareas of the ocular epithelium is documented. Parts of these results are confirmatory. In 2nd section, two migratory paths of Slc1a3+ cells were detected, a major radial one with cells migrating towards the cornea center, and a minor lateral pathway along the limbus border. By applying different chase periods and measuring distance of clones from the limbus border, it is indicated that some of the radial clones are faster cycling and shorter-lived, while the lateral ones appeared to divide extremely slowly. In 3rd section, it is documented that many Dlx1+, or K14+ cells are found in central cornea. They were found still after 3 months chase, but had mostly vanished after 1 year chase, which led the authors to postulate that these clones represented shorter-lived progenitor cells. Then, in 4th section, long-lived LRCs were detected to the fornix region, which appeared to not move away from there. The two markers for non-LRCs were also found in other distinct areas of the conjunctiva (bulbur or palpebral regions). The last section (5th) deals with effects after corneal injury. After limbus tissue deletion, Slc1a3+ cells, e.g., slowly cycling LRCs quickly were activated to replenish limbal tissue. After alkaline burns, Slc1a3+ cells migrated towards the cornea center, while K14+ cells from their particular conjunctival locations moved towards the limbus to possibly compensate for the cell loss there.

Discussion:
The Discussion section mostly is satisfactory. However, relevance of these findings from mice should be discussed as to their possible relevance for human eyes and vision diseases.

We appreciate the reviewer's insightful comments. Our study proposed possible heterogeneity within the limbus SCs and identified the previously uncharacterized Slc1a3^{CreER+} slow-cycling, laterally-expanding SC population. This limbal population is inactive during normal homeostasis, but it rapidly expands in response to injury to regenerate ocular surface epithelium, suggesting that these cells can be particularly useful after extensive ocular damages such as severe chemical burns or inflammatory diseases, e.g., Stevens-Johnson syndrome. Our genetic tools will contribute to further address SC dynamics in different disease or injury models in vivo and to find a potential therapeutic strategy for limbal SC deficiency. The epithelial SC heterogeneity may also be relevant for understanding the mechanisms of tumorigenesis and for in vitro reconstitution of 3D eye tissues in future. In the revised manuscript, we added discussion about the possible relevance of observed SC heterogeneity for clinical application in future (page 13, line 385 to page 14, line 394).

Major comments

7. To my frustration: Throughout this study, the K14 and Dlx1 markers are used to mark non-LRCs, while Slc1a3 is interpreted as to label LRCs. However, to my frustrating confusion, in 2nd para of Results this is stated vice versa (p. 6, upper): what´s wrong here? ...is it a typing or other language problem? ...do I get something wrong?

We are thankful for pointing this out. In our previous study in the mouse skin epidermis, Dlx1 and Slc1a3 markers were identified as LRCs and non-LRCs (page 5, lines 130-132). In contrast, Dlx1 and

© 2020. Published by The Company of Biologists under the terms of the Creative Commons Attribution License (https://creativecommons.org/licenses/by/4.0/).

Slc1a3 markers showed an opposite pattern in the ocular surface epithelium (Dlx1 in non-LRCs and Slc1a3 in LRCs). To avoid any confusion, we have carefully changed some descriptions in the revised manuscript (page 7, lines 174-177).

8. Check quantification procedure (statistics)?: I understand that all clones from 1 whole-mounted half eye are used for quantification, and this is done for 3 mice (= 3 half eyes quantification)? The data are combined and averaged? What happened with the second eye from each mouse? These details must be provided in M&M.

We are thankful to the reviewer's suggestion to clarify the quantification methods. We counted all clones from a half eye per mouse and repeated experiments with N=3 mice for $Slc1a3^{CreER}$ and $K14^{CreER}$. Due to low efficiency of Cre, we used N=3 to 7 mice for $Dlx1^{CreER}$. N numbers are shown in figure legends and methods in the revised manuscript. Data are shown as the average of an individual mouse with standard deviation (SD). We used 1 eye for K12 and 1 eye for K19 staining.

9. In Figures, spatial relations between whole-mounted sample and section shown below remains unclear.
Thank you for the reviewer's suggestion. In the revised figures, we revised the cartoon to clearly indicate how section and whole-mount samples are taken (new Fig. 1B). We added a yellow dotted square in the whole-mounted image to indicate the area shown in the section view (new Fig. 2E-H, Fig. 3E-H, Fig. 4A-D, Fig.4I-P). We also added the original Z-stack images for projection and ortho (section) view in supplemental figures to indicate the relationship between whole-mounted sample and section view (new Fig. S3A-H, Fig. S4, Fig. S6).

10. What do we learn from sections vs. pictures from whole-mounted sheets? Findings from sections remain nearly unnoticed throughout the text; if mentioned, a precise numbering is missing: give precise Fig. No., e.g. "Fig. 3C, left frame".

As per reviewer's suggestion, we renumbered each figure and referred individual panel in the text. We also included some descriptions in the revised manuscript to explain the interpretation of section results (page 7, lines 198-200).

11. Some interpretations of results are speculative, and do not belong in Results section; e.g., p. 11, lower para: this statement in Results is highly speculative.

We agree with the reviewer that our interpretation of the relationship between $K14^{CreER}$- and $Slc1a3^{CreER}$-derived clones was speculative. In the revised manuscript, we removed the discussion.

12. P. 14, upper, Discussion, 1st para: does this study really provide data on "cell division frequency"? (see also p. 8: type "..suggest the possible heterogeneity of the ocular epithelium regarding its cell division..."): wording "cell division dynamics" might be more appropriate.

We agree with the reviewer's suggestion. We have changed the word "cell division frequency" to "cell division dynamics" in the title and text.

Minor points:

- Labels in figures: where is basal, where apical in sections? Label.

Thank you for the reviewer's comment. All z-stack images were arranged as the basal on the bottom and the apical on the top. We labeled all section images with "BL" to indicate basal layer in the revised manuscript.

- Fig. 4: the many white triangles within pictures are confusing: are these necessary/helpful?

We agree with the reviewer's comment. We deleted all white arrowheads from Fig. 4.

- Fig. 1E, middle: use different line symbols for both features.

We have changed the colour of each line symbol.

© 2020. Published by The Company of Biologists under the terms of the Creative Commons Attribution License (https://creativecommons.org/licenses/by/4.0/).

- "IFE" in results: what is it? ...explain in text.

Thank you for the reviewer’s comment. IFE is the abbreviation of interfollicular epidermis of skin. Since this abbreviation is not common, we spelled out as interfollicular epidermis in the revised manuscript.

- P. 8: type ...behavior of the LRC population...

We revised the manuscript accordingly.

Reviewer #2 (Significance (Required)):

This study is relevant for basic ocular sciences, and, in particular, for biomedical issues of stem cell-based tissue repair after corneal injuries.

REFEREE'S CROSS-COMMENTING:

I have read the comments of referee 1: I agree with most of his suggestions. I feel that our two reviews of this work are highly compatible.

Second decision letter

MS ID#: DEVELOP/2020/197590

MS TITLE: Defining compartmentalized stem cell populations with distinct cell division dynamics in the ocular surface epithelium

AUTHORS: Ryutaro Ishii, Hiromi Yanagisawa, and Aiko Sada

ARTICLE TYPE: Research Article

Your response to the review and editor's comment and the revision of the manuscript are satisfactory. The manuscript has been accepted for publication in Development, pending our standard ethics checks.

© 2020. Published by The Company of Biologists under the terms of the Creative Commons Attribution License (https://creativecommons.org/licenses/by/4.0/).
